# Large-scale lipid analysis with C=C location and *sn*-position isomer resolving power

**DOI:** 10.1038/s41467-019-14180-4

**Published:** 2020-01-17

**Authors:** Wenbo Cao, Simin Cheng, Jing Yang, Jiaxin Feng, Wenpeng Zhang, Zishuai Li, Qinhua Chen, Yu Xia, Zheng Ouyang, Xiaoxiao Ma

**Affiliations:** 1 Department of Precision Instrument, State Key Laboratory of Precision Measurement Technology and Instruments, Tsinghua University, Beijing, 100084 China; 2 Department of Chemistry, Tsinghua University, Beijing, 100084 China; 3 Department of Chemistry, Purdue University, West Lafayette, IN 47907 USA; 4 Affiliated Dongfeng Hospital, Hubei University of Medicine, Shiyan, Hubei Province 442000 China

**Keywords:** Lipidomics, Lipids, Analytical chemistry, Bioanalytical chemistry, Mass spectrometry

## Abstract

Lipids play a pivotal role in biological processes and lipid analysis by mass spectrometry (MS) has significantly advanced lipidomic studies. While the structure specificity of lipid analysis proves to be critical for studying the biological functions of lipids, current mainstream methods for large-scale lipid analysis can only identify the lipid classes and fatty acyl chains, leaving the C=C location and *sn*-position unidentified. In this study, combining photochemistry and tandem MS we develop a simple but effective workflow to enable large-scale and near-complete lipid structure characterization with a powerful capability of identifying C=C location(s) and *sn*-position(s) simultaneously. Quantitation of lipid structure isomers at multiple levels of specificity is achieved and different subtypes of human breast cancer cells are successfully discriminated. Remarkably, human lung cancer tissues can only be distinguished from adjacent normal tissues using quantitative results of both lipid C=C location and *sn*-position isomers.

## Abstract (web summary)

Coupling photochemical derivatization with tandem mass spectrometry enables C=C-isomer resolved lipidomics. Here, the authors further develop this approach into a shotgun lipidomics workflow that allows simultaneous characterization of lipid C=C locations and *sn*-positions in complex biological samples.

## Introduction

Lipids, especially glycerophospholipids (GPs), are the building-blocks of cell membranes and play important biological roles, such as energy storage, cell adhesion and migration, signal transduction, and apoptosis^1–3^. An increasing number of researches have revealed a correlation between lipid alterations and various diseases due to lipid reprogramming^4–9^. Currently, mass spectrometry (MS) has become the method of choice for lipid analysis, offering label-free detection at high sensitivity and structural characterization capability^10–18^. However, large-scale lipid analysis with a comprehensive capability of revealing all levels of structure information still represents a significant analytical challenge for lipidomics^19–21^. GPs, for instance, have five levels in terms of structure information, including lipid class, fatty acyl identities, fatty acyl *sn*-positions, and C=C location/geometry (viz cis/trans) in the fatty acyl. Successful attempts for determining C=C locations^22–28^ in fatty acyls or their *sn*-positions^28,29^ have already been reported for MS analysis, enabling characterization of detailed structure moieties and identification of lipid structure isomers. An extremely useful feature offered by lipid isomer analysis, is the relative quantitation achieved at high precisions without requiring the use of lipid standards, which is not readily available^25,26^. Remarkably, our recent study demonstrated close correlation between the lipid C=C location isomer compositions and Type II diabetes^30^, which owes to a tighter regulation on lipid desaturation, allowing efficient elimination of interferences due to variations among samples.

Various methods have been explored for differentiating the lipid C=C location and *sn*-position isomers. Ozone-induced dissociation (OzID)^31–33^ and ultraviolet photodissociation (UVPD)^28^ have been used to determine both *sn*-positions and C=C locations in GPs. By coupling photochemical (Paternò-Bǜchi, PB) reaction with tandem MS (MS/MS), we have systematically demonstrated the qualitative and quantitative analysis of lipids with C=C specificity from complex biological samples^25–27,34,35^. PB reaction converts the C=C to an oxetane which can be preferentially fragmented by low-energy collision-induced dissociation (CID). Other approaches include ion mobility spectrometry (IMS)^36,37^ and alternative gas-phase ion activation^38–44^. These lipid characterizing methods were also compatible with mass spectrometry imaging (MSI)^45–50^. For instance, OzID implemented with matrix-assisted laser desorption ionization (MALDI) MSI revealed altered fractions of phosphatidylcholine (PC) *sn*-isomers in a tumorous mouse brain^45^. Similarly, MSI based on PB-MS/MS^34,46^ and UVPD^47^ showed the spatial distribution of lipid C=C location isomers in brain, thyroid, and breast cancer tissues, revealing significant fractional changes of C=C location isomers in cancerous tissues. These studies highlight the significance of developing lipid analysis methods with high structural specificities and their huge potentials in uncovering biological features invisible to conventional lipid profiling.

An ideal analytical tool for lipidomics in order to survey a wide range of lipids in discovery work should not only provide detailed information at multiple lipid structure isomer levels (e.g., C=C location/geometry and *sn*-position), but also be feasible for large-scale quantitative analysis. UVPD is capable of assigning C=C locations and *sn*-positions of fatty acyls, while OzID may be the only one that has been well demonstrated for assigning C=C locations in *sn*-specific fatty acyls. One problem of OzID is the long reaction time required for the ion trap implementation, however recent work has demonstrated the ability to perform OzID on LC-compatible time scales in the high pressure regions of the MS system^51^. For PB reaction method, both shotgun analysis^25,34^ and HPLC-PB-MS/MS workflow^30^ have been developed for identifying a large number of C=C location isomers.

In this work, we further develop the PB reaction method, to enable the *sn*-position specificity as an integrated feature into the simple and streamlined workflow for the large-scale, qualitative and quantitative analysis of lipids. Differential levels of lipid structure information, except for C=C geometry, now can be acquired in a single MS run. This is achieved by selecting the appropriate PB reagents that promotes the generation of diagnostic ions specific to both *sn*-positions and C=C locations, leading to confident structural elucidation and accurate relative quantitation. The method is validated by analyzing GPs from bovine liver and *Escherichia coli* (*E. coli*) via shotgun lipidomics, with 45 and 24 lipid structure (*sn*-/C=C) isomers identified. Quantitation of 87 identified lipid isomers is performed to distinguish four subtypes of human breast cancer cells, leading to a successful classification based on either C=C location or *sn*-position isomer compositions. This method is also applied to analyze lipids in type-2 diabetes (T2D) human plasma samples and significant changes in a set of PCs are identified at high confidence. The ultimate power of the enhanced structural specificity level is demonstrated by analysis of human lung cancer tissue samples, where cancerous tissues can be correctly distinguished from normal tissues ONLY via quantitative analysis of both GP *sn*-position and C=C location isomers. The developed methodology can be easily implemented for routine analysis, empowering the discovery of biomarkers with a tool revealing rich lipid structure details.

## Results

### Comprehensive structural elucidation of lipids by PB-MS^3^

**Fig. 1:**
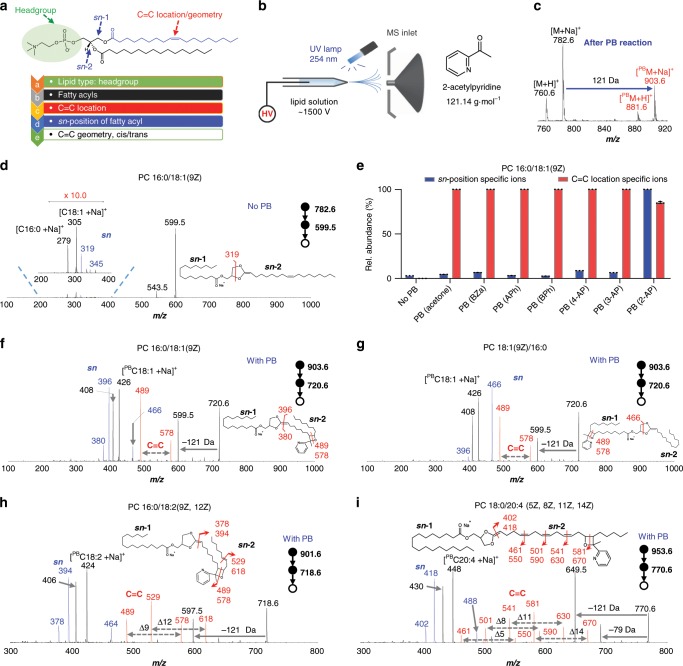
Comprehensive structure analysis of lipid C=C location and *sn-*isomers. **a** The chemical structure of PC 18:1(9Z)/16:0. **b** Schematic of the experimental setup for online derivatization of unsaturated GPs by coupling 254 nm irradiation with nanoESI-MS. **c** MS spectrum of PC 16:0/18:1(9Z) after 30 s reaction. **d** MS^3^ spectrum of PC 16:0/18:1(9Z) without PB derivatization. Sodiated lipid precursors were first fragmented to generate product ions after headgroup loss (−183 Da). Product ion at *m/z* 599.5 was further fragmented to release *sn*-1-specific diagnostic ions at *m/z* 319 (C16:0) and 345 (C18:1). **e** Comparison the relative abundance of *sn*-position and C=C location specific ions of PC 16:0/18:1(9Z) without and with PB derivatization using different PB reagents (Bza: benzaldehyde, APh: acetophenone, BPh: benzophenone, AP: acetylpyridine). Error bar represents the standard deviation, *n* = 3. **f**, **g** PB-MS^3^ spectra of PC 16:0/18:1(9Z) (**f**) and PC 18:1(9Z)/16:0 (**g**). Product ions at *m/z* 489 and 578 were C=C-specific diagnostic ions due to oxetane cleavage, indicated C=C at Δ9 in C18:1. Product ion at *m/z* 466 was specific for C16:0 at *sn*-2. Product ion at *m/z* 396 was specific for C18:1 at *sn*-2. **h**, **i** PB-MS^3^ spectra of PC 16:0/18:2(9Z, 12Z) (**h**) and PC 18:0/20:4(5Z, 8Z, 11Z, 14Z) (**i**). Refer to insets for detailed fragmentation pathways responsible for generating diagnostic ions. Peaks labeled in red and blue are C=C-specific and *sn*-specific diagnostic ions, respectively. Source data are provided in a Source Data file.

**Fig. 2:**
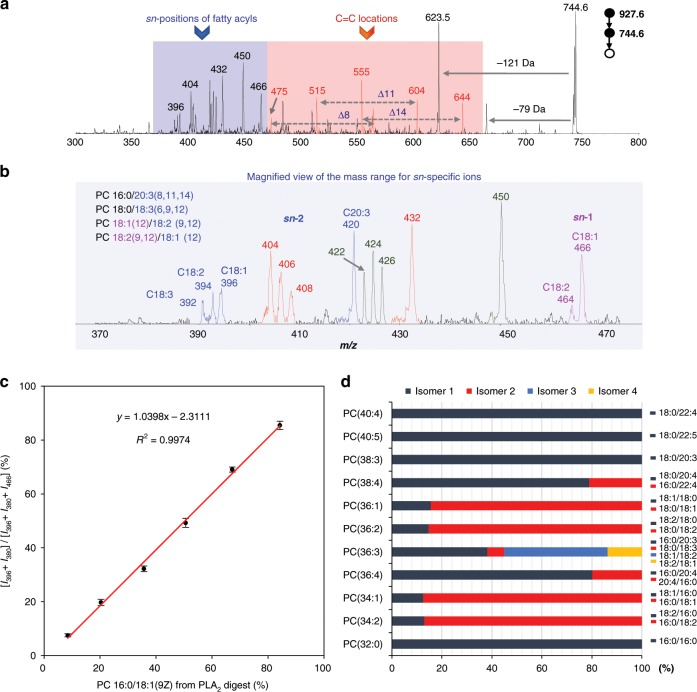
Simultaneous determination of GP C=C location and *sn-*isomers. **a** The MS^3^ spectrum of PC 36:3 in the extract after loss of headgroup by CID. C=C-specific diagnostic ions were labeled in red, while ions reporting the *sn*-positions of fatty acyls were shaded in blue. **b** A magnified view of the shaded in blue *m/z* region showing the set of fatty acyls at *sn*-2 position including C18:1, C18:2, C18:3, and C20:3 (labeled in blue), the set of fatty acyls at *sn*-1 position including C18:1, and C18:2 (labeled in purple), sodiated PB products of FA 18:1, FA 18:2, FA 18:3, and FA 20:3 at *m/z* 426, 424, 422, and 450 (labeled in dark green) and the corresponding H_2_O loss peaks (labeled in red). **c** Correlation between the PLA_2_ digestion and PB-MS^3^ methods for the relative quantitation of PC 16:0_18:1 *sn*-isomers. Error bar represents the standard deviation, *n* = 3. **d** The relative composition of *sn-*isomers of GPs. The relative amount was calculated by the intensity of the *sn*-specific diagnostic ions for a specific isomer divided by the total intensity of those for all possible *sn*-isomers. For detailed formula, see (Supplementary Fig. 13). Source data are provided in a Source Data file.

The use of photochemistry and tandem MS for lipid structure analysis have been systematically studied on both nano-electrospray ionization (nanoESI)-MS^25,34^ and LC-MS platforms^30^. All these studies used acetone as the reactant owing to its miscibility with water and other commonly used organic solvents, high reaction kinetics and compatibility with electrospray ionization. However, as much suitable for large-scale as it is, this strategy has not been shown to be capable of determining the *sn*-positions of fatty acyls, another level of important lipid structures. Our previous study revealed elevated levels of C18:1(9) in GPs in human and mouse breast cancer tissues, suggesting the potentials of lipid isomer analysis to discover biomarkers for disease diagnosis. The enhancement in the structural specificity to include the *sn-*position resolving power into the workflow would significantly improve its capacity serving as an analytical tool for biological studies. While using the PB reaction method for lipid analysis, we accidentally observed that some fragment ions specific to the *sn*-positions were produced during the CID of the PB product ions. As shown in Fig. 1f, for analysis of PC 16:0/18:1(9Z) standard, *m/z* 489 and *m/z* 578 were diagnostic ions for the C=C location while *m/z* 396 and *m/z* 466 could serve as diagnostic ions for C18:1 at *sn*-2 and C16:0 at *sn*-2. A systematic screening was performed for a group of carbonyl compounds^52,53^ (Supplementary Fig. 7 and Supplementary Table 3) using the nanoESI-MS platform shown in Fig. 1b. It was found that 2-acetylpyridine (121.14 g∙mol^−1^) was the most suitable reagent for enhancing the intensities of the *sn*-position diagnostic ions. This reagent was used as the PB reagent for identifying the C=C locations of fatty acids, fatty acid esters, cholesterol esters, triglycerides, and unsaturated hydrocarbons^54^. In this study, we found it particularly suitable to serve our purpose due to the following advantageous features: (1) diagnostic ions of high abundance produced for unambiguous identification of C=C locations and *sn*-positions (Fig. 1e); (2) adequate or high PB reaction yield could be obtained; (3) compatible with electrospray ionization (ESI) for shotgun lipidomics; (4) increase of *m/z* value over 100 Da for PB reaction products which simplify the spectral complexity by eliminating the overlapping of products with intact GPs. In addition, no instrumental modification to the MS instrument was needed. At a molar ratio of 1/100 (lipid/2-acetylpyridine), the reaction yield reached ~35% after only 30 s of UV irradiation (254 nm, Supplementary Fig. 1). No major side reaction (Norrish type I&II) products were detected (Fig. 1c).

We initially validated the developed method using two pairs of PC *sn*-isomers of PC 16:0_18:1 and PC 18:0_18:1. Without any photochemistry, MS^3^ analysis enabled discrimination of *sn*-isomers. For PC 16:0/18:1, intact lipids first underwent a headgroup loss (−183 Da) to produce dioxolane bonded to the fatty acyls (*m/z* 599.5) (Supplementary Fig. 2). The *sn*-2 fatty acyl was attached to dioxolane via a newly formed C=C. Further CID of the dioxolane induced cross-ring cleavage to produce a fragment at *m/z* 319, specific to C16:0 at *sn*-1 (Fig. 1d). However, we also detected a less abundant ion at *m*/*z* 345, suggesting C18:1 at *sn*-1 that is inconsistent with the PC structure (Fig. 1d). Similar results were observed for PC 18:1/16:0 and *sn-*isomers of PC 18:0_18:1 (Supplementary Figs. 4 and 5). This could either be due to isomeric impurity as reported in previous studies^28^, or the non-specificity of fragment ions *m/z* 319 and 345 to *sn*-positions. To clarify this ambiguity, a regiopure triacylglyceride (TAG), TAG 18:1(9Z)/16:0/18:1(9Z) was analyzed to afford a similar dioxolane structure at *m/z* 599.5, after loss of one C18:1 chain. No matter which C18:1 was lost, C16:0 was always at *sn*-2 position. CID of the dioxolane produced the ion *m/z* 345 exclusively (Supplementary Fig. 6a), which thus confirmed the *sn*-specificity of ions produced following cross-ring cleavage.

Having confirmed that MS^3^ analysis of sodiated GPs can be used for resolving *sn*-positions of fatty acyls, we set to explore whether this capability can be integrated into PB-MS/MS for simultaneous determination of C=C locations within and *sn*-positions of fatty acyls in GPs. For method validation, we chose a pair of PC *sn*-isomers, i.e., PC 16:0/18:1 and PC 18:1/16:0 (10 μM for each). The same experimental workflow was followed by analyzing sodiated PB products of GPs (Fig. 1f, g). The *sn*-positions of fatty acyls were indicated by *sn*-specific ions at *m/z* 396 and 380, owing to cleavage at the newly formed C=C after the 183 Da loss. A possible fragmentation pathway for dioxolane formation via a concerted fragmentation mechanism^55^ were proposed in Supplementary Fig. 8b. Product ions at *m*/*z* 489 and 578 were identified to be C=C-specific diagnostic ions, indicating cleavage at Δ9 C=C in C18:1 (Supplementary Fig. 3). Fragments related to unsaturated fatty acyl were detected only, owing to high sodium affinity of C=C-fused pyridine^56^. The product ion at *m/z* 466 (indicating C18:1 at *sn*-1) was due to cross-ring cleavage of the dioxolane (Supplementary Fig. 8a). The fragmentation schemes of the dioxolane were distinct between C18:1 at *sn*-1 and *sn*-2 positions (*m/z* 396 vs. 466), which is the underlying mechanism for pinpointing *sn*-positions of fatty acyls. PB-MS^3^ analysis of regiopure TAG 18:1/16:0/18:1 revealed a *sn*-2 C16:0 chain exclusively, confirming the *sn*-specificity of the method (Supplementary Fig. 6b). The identity of the FA moiety doesn’t affect the *sn*-specificity (Supplementary Fig. 6c, d). Compared with MS^3^ spectrum of intact GPs (Fig. 1d), after PB reaction the relative intensity of *sn*-specific diagnostic ion increased by ~20 folds (Fig. 1f). The overall increase in detection sensitivity based on *sn*-specific diagnostic ions was ~10 folds, after accounting for ~20% derivatization yield, which is highly beneficial for analyzing lipids of low abundance.

We also evaluated the efficacy of the method for PCs with multiple degrees of unsaturation, using PC 16:0/18:2(9Z, 12Z) and PC 18:0/20:4(5Z, 8Z, 11Z, 14Z) as two examples. The abundant *sn*-specific ions at *m/z* 394 and 418 were consistent with C18:2 and C20:4 at *sn*-2 positions (Fig. 1h, i), clearly suggesting that our method is widely applicable to GPs containing one or multiple C=Cs. C=C-specific diagnostic ions were also detected with high abundance (Fig. 1h, i). Besides, the method can also be applied to other GP subclasses, such as phosphatidylethanolamine (PE), phosphatidylserine (PS), phosphatidylglycerol (PG), phosphatidylinositol (PI), and phosphatidic acid (PA) (Supplementary Fig. 9). The formation efficiency of the headgroup-loss MS^2^ fragment for each lipid class was listed in Supplementary Table 5. The limit of detections (LODs) for C=C and *sn*-position assignment were 5 nM for PC, 10 nM for PE, 40 nM for PS, 35 nM for PG, 50 nM for PA, and 50 nM for PI (Supplementary Fig. 10). Our workflow was also demonstrated to be compatible with the LC-MS workflow (Supplementary Note 1 and Supplementary Fig. 11).

### Analysis of GP isomers in bovine liver and *E. coli* extracts

The lipidome of mammalian cells or bacteria has a plethora of *sn*- and C=C location isomers. To demonstrate a comprehensive analysis of lipid *sn-* and C=C location isomers in complex biological samples, we analyzed lipid extracts from bovine liver and *E. coli*. The relatively large 121 Da mass increase in GPs helps to avoid overlapping of derivatized GPs with intact ones (Supplementary Fig. 12). In the bovine liver extract, 24 PC structural isomers were identified, including 16 *sn-*isomers, 8 C=C location isomers, and 5 fatty acyl composition isomers, e.g., PC 18:0/18:3 and PC 16:0/20:3 (Supplementary Table 6). C18:1 in all GPs contained Δ9 (major) and Δ11 (minor) isomers. However, in some species (e.g., PC 18:1_18:2), an additional Δ12 isomer was observed. Moreover, in addition to PCs, lipid isomers of other GP subclasses, including PE, PS, and PI, were also detected and identified (Supplementary Table 6 and Supplementary Fig. 14). In the *E. coli* extract, 24 PE isomers were identified, including 20 *sn-*isomers and 8 fatty acyl composition isomers (Supplementary Table 7). Interestingly, the C=C in C18:1 in all GPs is at Δ11 exclusively^57,58^. This phenomenon can be explained by the different molecular mechanisms of lipid desaturation and elongation in *E. coli* from eukaryotes^59,60^.

A remarkable feature of our method is that both lipid *sn*-isomers and C=C location isomers can be resolved from a single spectrum. Using PC 36:3 as an example, *sn*-specific ions at *m/z* 420, 396, 394, and 392 indicated C20:3, C18:1, C18:2, and C18:3 at *sn*-2 (Fig. 2a, b, Supplementary Note 2 and Supplementary Fig. 17). While the total carbon number and degrees of unsaturation (36 and 3) can be determined from precursor mass, once *sn*-2 fatty acyls were determined, *sn*-1 fatty acyls can be deduced easily (in fact *sn*-1 C18:2 and C18:1 were detected at *m/z* 464 and 466). Therefore, PC 36:3 can be confidently characterized as a mixture of PCs 16:0/20:3, 18:0/18:3, 18:1/18:2, and 18:2/18:1. Consistent with the three C=Cs, we detected three pairs of diagnostic ions at *m/z* 475/564, 515/604, and 555/644. C=C location(s) in each unsaturated fatty acyl can be determined accordingly. Similarly, PE 36:2 from *E. coli* extract was a mixture of PE 18:0/18:2(8, 11), PE 18:2(8, 11)/18:0, PE 18:1(11)/18:1(11), and PE 16:0/20:2(10, 13) (Supplementary Fig. 15). The structure-resolving capability for GPs at both *sn*-position and C=C location levels represents a near-complete lipid structure characterization except for C=C geometry, from only a single MS spectrum.

To evaluate the hypothesis of using *sn*-specific ions to quantify GP *sn*-isomers, a pair of PC 16:0_18:1 *sn*-isomers mixed at different molar ratios was analyzed. The quantitative accuracy of our method was benchmarked against a well-established method, i.e., phospholipase A_2_ (PLA_2_) digestion followed by quantitation of lyso-PC species. The molar percentage of PC 16:0/18:1(9Z) determined by PLA_2_ assay was plotted against the intensity percentage of its *sn*-specific ions (*m/z* 380 and 396) (Fig. 2c, *y* axis). The PB-MS^3^ results correlated extremely well with the PLA_2_ assay with a *R*^2^ of 0.9974. The regioisomeric impurities in PC 16:0/18:1 and PC 18:1/16:0 standards were 15% and 8% (cf. *sn*-specific ions%), which is consistent with previous studies^28,61,62^. Besides, we also validated the *sn*-specificity of our method using PC 18:0/20:4 and other classes of phospholipids, and all PB-MS^3^ results were highly consistent with PLA_2_ assays (Supplementary Table 4). The relative amounts of PC and PE *sn*-isomers in bovine liver polar extract and *E. coli* extract were listed in Fig. 2d and Supplementary Fig. 15c. The qualitative and quantitative analysis of GP C=C location and *sn-*isomers in one single experiment greatly simplifies the experimental procedure while boosts sample economy.

### Assignment of C=C location to individual fatty acyls in GPs

**Fig. 3:**
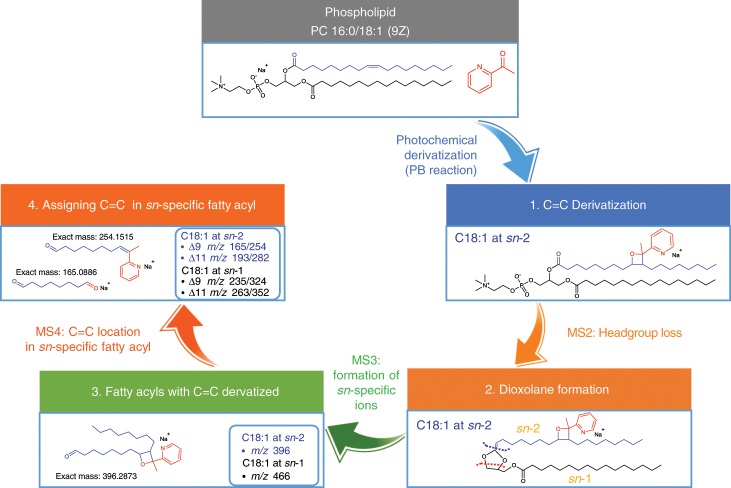
Assignment of C=C location within a *sn-*specific fatty acyl. General analytical workflow for assigning C=C location(s) in individual fatty acyls at specific *sn*-positions in GPs.

Currently, the assignment of C=C location to individual fatty acyls at specific *sn*-positions was only possible through CID/(OzID)^2^ or (CID/OzID)^2^. We aim to achieve the same level of lipid structure analysis using the developed method. Using PC 16:0_18:1 and PC 18:0_18:1 from bovine liver as model lipids, lipid PB products first lost the headgroup (183 Da) via CID for dioxolane formation. MS^2^ ions were fragmented to release *sn*- (*m/z* 380/396 and 466 for C18:1 at *sn*-2 and *sn*-1, respectively) and C=C location specific ions. To assign C=C location in individual fatty acyls, *sn*-specific ions were further isolated for generating C=C location specific ions (Fig. 3). As expected, MS^4^ analysis of PC 16:0_18:1 from bovine liver showed that C18:1 at both *sn*-positions was composed of Δ9 or Δ11 isomers (Supplementary Fig. 16a, b), and it seemed there was no preference for the incorporation of C18:1 C=C location isomers at different *sn*-positions. Similar results were acquired for PC 18:0_18:1 from bovine liver (Supplementary Fig. 16c, d).

### Characterization of human breast cancer cell lines

**Fig. 4:**
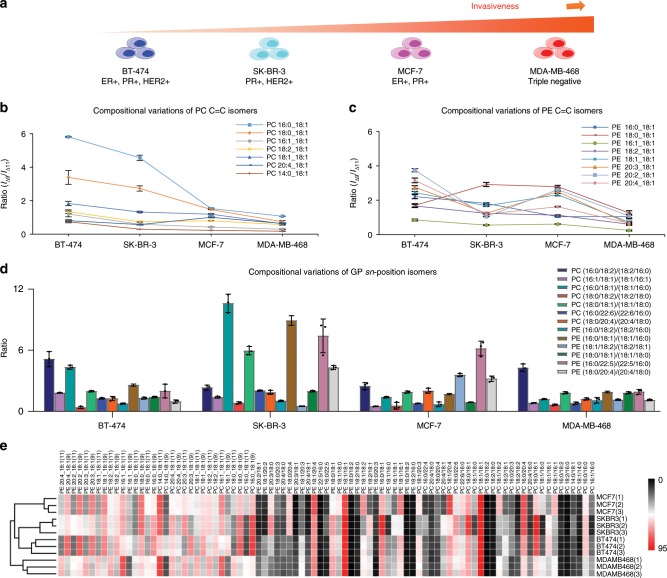
Deep lipidomics analysis of human breast cancer cell lines. **a** Four subtypes of human breast cancer cells (i.e., MCF-7, SK-BR-3, MDA-MB-468, and BT-474). Compositional variations of (**b**) PC C = C location isomers and (**c**) PE C = C location isomers among the four different breast cancer cell lines. Error bar represents the standard deviation, *n* = 3. **d** Compositional variations of GP *sn*-isomers among the four different breast cancer cell lines. Error bar represents the standard deviation, *n* = 3. **e** Hierarchical cluster analysis discriminated the four subtypes of human breast cancer cells by quantitative analysis of GPs at two isomer levels, i.e., *sn*-position and C=C location. Colors represent relative amounts as indicated by the color bar. Source data are provided in a Source Data file.

In this section, we aim to differentiate four different subtypes of human breast cancer cells (i.e., MCF-7, SK-BR-3, MDA-MB-468, and BT-474) by monitoring the compositional variations of GP C=C location and *sn*-position isomers. Conventional classification of these four subtypes is based on the expression of ER (estrogen receptor), PR (progesterone receptor), and HER2 (human epidermal growth factor receptor 2) (Supplementary Table 8). Using a shotgun lipidomics approach, we have identified a total of 87 GP isomers by mapping detailed lipid structures, of which 32 were C=C location isomers and 55 were *sn*-position isomers. For more than 75% of these GP isomers, their relative amounts were observed to show significant differences (*p* *<* 0.05) among the four different breast cancer cell lines (Supplementary Tables 9–11). At the C=C location isomer level, for instance, C18:1(9)-containing PCs, such as PC 14:0_18:1, PC 16:1_18:1, and PC 20:3_18:1 were elevated in BT-474 cells (*p* *<* 0.001) (Fig. 4b). By contrast, C18:1(11)-containing PEs, such as PE 16:1_18:1, PE 18:2_18:1, PE 18:1_18:1, PE 20:3_18:1, and PE 20:4_18:1, were elevated in MDA-MB-468 cells (*p* *<* 0.001) (Fig. 4c). At the *sn*-position isomer level, PC 16:0/18:1 in PC 34:1, PC 18:0/18:1 in PC 36:1, PC 16:0/22:6 in PC 38:6, PE 16:0/18:1 in PE 34:1, and PE 18:0/20:4 in PE 38:4 were elevated in SK-BR-3 cells (*p* *<* 0.001). Increased amounts of most PC and PE isomers with a more unsaturated fatty acyl at *sn*-2 position were detected (Fig. 4d).

The observed variations in the compositions of GP isomers enabled successful discrimination of all four subtypes of breast cancer cells by hierarchical cluster analysis (HCA) either at the C=C location isomer level or *sn*-position isomer level (Supplementary Figs. 18–20). In particular, the clusters between MDA-MB-468 and other cell lines exhibited the longest distance (Fig. 4e), indicating a most pronounced difference in its GP isomer composition. Consistently, we observed drastically elevated C18:1(11)-containing GPs in almost all abundant lipid species in MDA-MB-468 cells (Fig. 4b, c). In fact, the relative amounts of C18:1(11)-containing GPs gradually increased in the sequence of BT-474 < SK-BR-3 < MCF-7 < MDA-MB-468 (Fig. 4a), which is consistent with invasiveness potential of breast cancer cells^63^. Interestingly, the four subtypes of breast cancer cells could also be successfully differentiated using C=C information specific for individual *sn*-positions (Supplementary Fig. 21). These subtypes could also be discriminated by principal component analysis (PCA) (Supplementary Fig. 22). In addition, we observed that the relative standard deviations (RSDs) were ≤15% and ≤10% for quantifying lipid *sn*- and C=C location isomers, respectively.

### Analysis of human type-2 diabetes plasma samples

**Fig. 5:**
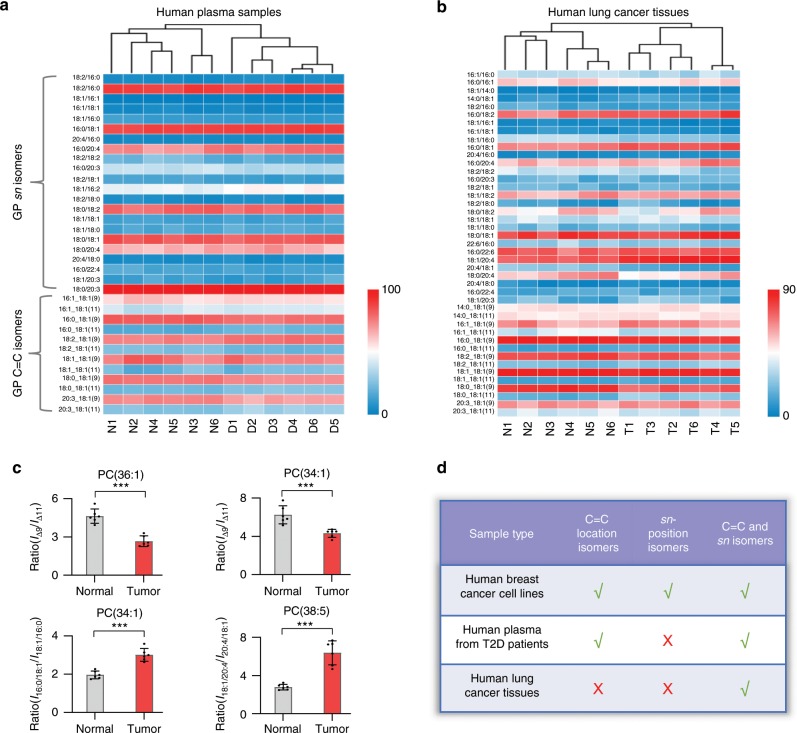
Analysis of human plasma and lung cancer samples. **a** Hierarchical cluster analysis of normal (N1-N6) and T2D (D1-D6) human plasma samples using 22 *sn-*position and 12 C=C location isomers. **b** Hierarchical cluster analysis of paired human NSCLC (T1-T6) and adjacent normal tissues (N1-N6) using 29 *sn-*isomers and 14 C=C location isomers. Colors represent relative amounts as indicated by the color bar. **c** The C=C/*sn-*isomer ratios for representative PCs (PC 34:1, PC 36:1, and PC 38:5) in paired cancerous and normal human lung tissues. Error bar represents the standard deviation, *n* = 6. ***p < 0.001 (Student’s two-tailed *t*-test). **d** Summary of the cluster analysis to obtain correct discrimination of different samples in this study, by using either C=C location isomers or *sn-*isomers, or combing them together. Source data are provided in a Source Data file.

The quantitative analysis of C=C and *sn-*isomers of lipids offers more details on the complexity of the lipidome that is desirable for biomedical analysis. As a first example, we analyzed the lipid extracts of 6 normal (as control) and 6 T2D human plasma samples. To classify normal and diseased samples, 22 *sn-*isomers and 12 C=C location isomers (containing C18:1, Δ9/Δ11) were analyzed and quantitated. Combining C=C-specificities and *sn*-specificities, the 12 plasma samples were correctly classified using HCA (Fig. 5a). Using *sn*-specificity only led to wrong results (Supplementary Fig. 25). Relative C=C isomer ratios were measured for 6 pairs of Δ9/Δ11 isomers of C18:1-containing PCs, among which four pairs exhibited significant changes (*I*_Δ9_/*I*_Δ11_, *p* *<* 0.001, two-tailed Student’s *t* test) in C=C isomer composition between T2D and normal plasma samples, including PC 16:0_18:1, PC 18:2_18:1, PC 18:0_18:1, and PC 20:3_18:1 (Supplementary Fig. 26). Distinct *sn*-position isomers were also identified to exhibit significant changes in isomer compositions between T2D and normal plasma samples, such as PC 36:3, PC 36:4 (*p* *<* 0.001), and PC 38:4 (*p* *<* 0.001) (Supplementary Fig. 27).

### Analysis of paired normal and cancerous human lung tissues

We aim to discriminate non-small cell lung cancer (NSCLC) tissues from adjacent normal tissues through lipid structure isomer analysis. Changes in the compositions of lipid isomers are indicators of altered stearoyl-CoA desaturase (SCD) or phospholipase activities^59^. Existing studies have shown that cancer cells in solid tumors are heterogeneous, which could lead to large variations in lipid compositions of tissues from different patients^64^. Among the total 43 PC structure isomers identified, 29 were *sn-*isomers and 14 were C=C location isomers. However, few pairs of *sn-*isomers showned statistically significant changes in isomer composition in NSCLC tissues (*n* = 6). The *sn*-isomer composition *I*_(16:0/18:1)/(18:1/16:0)_, defined as the intensity ratio between *sn-*specific ions for PC 16:0/18:1 and those for PC 18:1/16:0, increased from 2.0 ± 0.2 to 3.0 ± 0.3 (*p* *<* 0.001); while *I*_(18:1/20:4)/(20:4/18:1)_ increased from 2.8 ± 0.3 to 6.3 ± 1.0 (*p* *<* 0.001) (Supplementary Fig. 29). By contrast, the relative amount of PC 18:0/20:4 in PC 18:0_20:4 decreased (Supplementary Fig. 30). No significant changes in *sn*-position isomer composition were observed for other abundant PC species, including PC 34:2, 36:1, 36:2, 36:4, 38:4, and 38:6 (Supplementary Fig. 31). HCA using GP *sn-*isomers or C=C location isomers failed to discriminate cancer tissues from normal tissues (Supplementary Fig. 33). To our delight, with both C=C-specificities and *sn*-specificities, all 12 tissues can be correctly classified (Fig. 5b). Of the 14 C=C location isomers, the relative amounts of C18:1(9)-containing isomers were decreased in PC 34:1, PC 36:1, and PC 36:2 in cancer tissues (Fig. 5c and Supplementary Fig. 32). No statistically significant changes in the relative amounts of other C=C location isomers were observed. Importantly, as expected variations in *sn-*isomer compositions for GPs were very stable as well (RSD < 20%, *n* = 6, Supplementary Fig. 28). PCA using both types of isomers was also successful in tissue classification, and lipid species with major contributions were identified (Supplementary Fig. 34). Therefore, we provide strong experimental evidence that comprehensive lipid analysis at both C=C location and *sn*-position levels led to improved analytical performance in biomedical analysis (Fig. 5d).

## Discussion

In this study, we report a one-step method that allows a large-scale and high structure specificity identification of lipids in complex mixtures. Compared with the absolute intensities of diagnostic ions prior to PB reaction, PB derivatization using 2-acetylpyridine has led to ~20 folds increase in the absolute intensities of *sn*-specific ions (Supplementary Tables 1 and 2). No C=C-specific ions can be detected without PB derivatization. The method was also evaluated on a mass spectrometer at a mass resolution of 60,000 (Supplementary Note 3, Supplementary Fig. 35 and Supplementary Table 12). The preferential generation of highly abundant diagnostic ions simultaneously for *sn*-positions and C=C locations greatly simplified spectra interpretation. Identification and quantitation of lipid *sn*- and C=C location isomers were achieved in a single experiment, without using lipid standards. With a simple experimental workflow that can be easily coupled with shotgun lipidomics, high abundance of both *sn*-specific and C=C-specific ions were produced. Previously, we have reported significantly increased analytical precision in biological analysis by analyzing GP C=C location isomers, owing to the self-correction in lipid ionization frequently perturbed by ion suppression, matrix effects, and circadian rhythm, et al. In this work, we found a comparable analytical precision for lipid isomer analysis at both *sn*-position and C=C location levels, which further improved the distinguishing capability of our method for analyzing diseased samples, besides the more comprehensive interpretation of lipidome complexity. Identification and quantitation of lipids with C=C and *sn*-specificities is achieved by a single PB-MS^3^ workflow. The LODs for analyzing lipid isomers of different classes were determined to be comparable to those via conventional lipid analysis with no isomer-resolving power (Supplementary Table 5). We anticipate that a complete lipid structure characterization, including C=C geometry, will ultimately expand lipid structure isomer analysis to even more levels and lead to robust biological analysis tools.

Continuous method development constantly removes barriers for lipid characterization by elucidating lipid structures with increased details, such as C=C location within and *sn*-positions of fatty acyls. This paves the way for detecting and quantifying structurally defined lipids, and to further investigate their biological functions. Quantitation of structurally resolved lipids also has huge implications for disease diagnosis, as demonstrated in the discrimination of T2D human plasma and cancer tissues using multiple types of lipid isomers. The addition of more detailed lipid analysis, e.g., lipid isomer resolving, should lead to more accurate analytical and diagnostic tools. The observed changes in lipid isomer compositions in diseased states potentially indicate altered expression or activities of enzymes involved in lipid metabolism. In human cells, for instance, the biosynthesis of mono-unsaturated fatty acids (MUFAs) was catalyzed by two SCDs, i.e., SCD1 and SCD5. SCD1 introduces a C=C into C16:0 or C18:0 to produce C16:1(9) or C18:1(9), and C16:1(9) can be further elongated to C18:1(11) by FA elongases (Elovl-1,3,6). By contrast, SCD5 has a distinct substrate specificity to catalyze the desaturation of C18:0 to C18:1(9) only (Supplementary Fig. 23). To gain insights on the relationship between relative amounts of C18:1 C=C location isomers and SCD1 activity, we inhibited SCD1 in MCF-7 cells via a small-molecule inhibitor (CAY10566)^65^ and detected elevated amounts of C18:1(9)-containing GPs as expected (Supplementary Fig. 24). This finding possibly offers an alternative way to probe lipid desaturation. A large body of studies have consistently reported the elevated levels of SCD1 in cancers^66^. In summary, our method can serve as an enabling tool not only to tackle the structural complexity of the lipidome but also to accurately monitor the differential expression of lipid-related enzymes to ultimately identify therapeutic vulnerabilities.

## Methods

### Sample preparation

Lipid standards and bovine liver polar extract were purchased from Avanti Polar Lipids (Alabama, USA). Carbonyl compounds were purchased from J&K (Beijing, China). Phospholipase A_2_ from *Crotalus adamanteus* venom was purchased from Aladdin (Shanghai, China). Sodium acetate was purchased from Sinopharm (Beijing, China). All other chemicals (HPLC-grade) were purchased from Fisher Scientific (NJ, USA) and used without further purification. Lipid stock solutions were prepared in chloroform or methanol. Human type-2 diabetes (T2D) plasma samples and non-small cell lung cancer (NSCLC) tissue samples were supplied by Dongfeng Hospital of Hubei University of Medicine. All the procedures related to these samples were compliant with all relevant ethical regulations set by the Ethical Review Board of Tsinghua University (IRB No. 2017007). Informed consent was obtained from all participants.

Human breast cancer cell lines including MCF7 (catalog number 3111C0001CCC000013), SK-BR-3 (catalog number 3111C0001CCC000085), BT-474 (catalog number 3111C0001CCC000129) and MDA-MB-468 (catalog number 3111C0001CCC000249), were obtained from National Infrastructure of Cell Line Resource (Beijing, China). MCF-7 and SK-BR-3 cells were cultured in Dulbecco’s modified Eagle’s medium supplemented with 10% fetal bovine serum (FBS) and 1% penicillin-streptomycin. BT-474 cells were cultured in Roswell Park Memorial Institute-1640 medium supplemented with 10% FBS and 1% penicillin-streptomycin. MDA-MB-468 cells were cultured in Leibovitz’s L-15 medium with the same supplements. The cells were cultured with sealed culturing dish and the rest of cells were cultured with breathable dish in a humidified atmosphere containing 5% CO_2_ at 37 °C and passaged every 2 or 3 days. After reaching confluence, the cells were detached using 0.1% trypsin solution and collected by centrifugation. The cells were washed with phosphate-buffered saline PBS (2 mL). Then, methanol (1 mL) was then immediately added to quench the cells. Cell remnants were scraped from the culture dishes and collected in fresh tubes along with the cell lysates.

A modified Folch method was employed for lipid extraction from human plasma and tissue samples. For lipid extraction from plasma samples, 50 μL plasma was diluted by 1 mL deionized water in a 10 mL centrifuge tube, followed by an addition of 1 mL methanol and 2 mL chloroform. After 5 min of vortex, the mixture was centrifuged at 10,000 × *g* for 10 min (Eppendorf, Shanghai, China). The lipid extraction procedure was repeated once. The chloroform layers from two extractions were combined and dried under nitrogen flow. The dried lipid extract was stored at −20 °C before MS analysis. For lipid extraction from human tissue samples, 30–50 mg tissue sample was placed in a 10 mL centrifuge tube added with 1 mL of deionized water. The tissue sample was homogenized by a handheld homogenizer (Jingxin Technology, Shanghai, China) at 40,000 Hz for 5 min and subsequently mixed with 1 mL methanol and 2 mL chloroform to start liquid–liquid extraction. After 5 min of vortex, the homogenized tissue was centrifuged at 10,000 × *g* for 8 min. The bottom layer was collected and transferred to a 10 mL glass test tube. The above lipid extraction procedure was repeated once. The chloroform layers from the two extractions were then combined and dried under nitrogen flow. The extract was stored at −20 °C before MS analysis. The cell samples were extracted using a modified Folch procedure. In brief, chloroform (2 mL) and water (1 mL) were added to the cell tube. The mixture was then sonicated in a water bath for 10 min. After 30 s vortex, the mixture was centrifuged at 10,000 × *g* for 10 min. The extraction was repeated once. The chloroform layers from the two extractions were combined and dried under nitrogen flow. All extracts were stored at −20 °C before analysis.

A wild-type strain of *E. coli* (LM 3118) and Luria broth were purchased from Maojie Ltd. (Nanjing, China). The *E. coli* was grown in Luria broth at 37 °C on a rotary shaker (20 mL culture) overnight. The medium was prepared in distilled water and autoclaved under standard conditions. Then, cells were harvested by centrifugation at 10,000 × *g* for 10 min The pellet was recovered and dispersed in 1 mL deionized water. Lipids were extracted according to the modified Folch method. Firstly, 2 mL of CHCl_3_/CH_3_OH (2:1, v/v) was added to 1 mL bacterial suspension. The mixture was then vortexed, centrifuged at 1000 × *g* for 10 min The chloroform-rich phase at the bottom containing GPs was then removed with a Pasteur pipette. The extraction step was repeated twice and the combined organic phase was evaporated and the extract was stored at −20 °C before analysis.

### PLA_2_ digestion

The PLA_2_ digestion was performed using the protocol of Ekroos et al. with some modifications^61^. Briefly, we prepared a series of PC 16:0_18:1 solutions containing varied amounts of PC 16:0/18:1 (0, 20, 40, 60, 80, and 100%, molar percentage) at a total concentration of 10 µM. Each mixture (1.2 mL) was divided into three equal aliquots of 400 µL. One aliquot was mixed with 200 µL aqueous solution containing 20 mM Tris-HCl, 40 mM CaCl_2_ and 7 µg PLA_2_. The resulting mixture was vortexed for 2 min, and then incubated at 37 °C for 4 h. Lipids were extracted by adding 500 µL chloroform. The mixture was again vortexed for 2 min and centrifuged at 10,000 × *g* for 3 min The bottom phase was transferred into a glass vial and dried under nitrogen. Dried samples were reconstituted in methanol with 1% formic acid for MS analysis by monitoring the relative amounts of the lyso-lipids produced by PLA_2_ digestion via precursor ion scan (PIS) of the ion at *m/z* 184. Other types of lipids, including PE, PS, PG, PA, and PI, were analyzed by the same protocol. The lyso-PEs and lyso-PSs were monitored via neutral losses can (NLS) of 141 Da and 185 Da in positive mode, respectively. The lyso-PGs and lyso-PAs were monitored via PIS of the ion at *m/z* 153, while lyso-PIs were monitored via NLS of 241 Da in negative mode.

### MS-based shotgun lipidomics analysis

GP standard solutions of 10 µM and 20 μg/mL for bovine liver polar extract were prepared. Samples were dissolved in acetonitrile/water 50/50 (v/v) containing 1 mM 2-acetylpyridine. To minimize the formation of other adducts and promote the formation of sodium adducts, 100 μM sodium acetate was added to lipid solutions. The yield of sodium adducts from bovine liver polar extract was >85% (Supplementary Fig. 12). The commercial ESI source was removed and replaced with a homebuilt nanoESI source. The nanoESI capillary was made from a borosilicate glass capillary (1.5 mm o.d. and 0.86 mm i.d.) using a P-1000 Flaming/Brown micropipette puller (Sutter Instrument, Novato, USA). A stainless-steel wire was inserted into the nanoESI capillary to deliver high voltage for ESI. The nanoESI tip was aligned with the sampling orifice of the mass spectrometer. A low-pressure mercury (LP-Hg) lamp with emission centered around 254 nm (Model No.: 80-1057-01, BHK, Inc., CA, USA) was placed 1.0 cm away from the nanoESI emitter to initiate PB reactions. MS^3^ experiments were performed with CID sequentially by first isolating the desired [M + Na]^+^ species. The headgroup loss fragment ions generated from MS^2^ were subsequently isolated for MS^3^ analysis. Typically, 10–60 scans were acquired and averaged to obtain mass spectra with satisfactory signal-to-noise ratios. For the screening of PB reagents, the spray solutions are acetone/water 50/50 (v/v) for acetone, or acetonitrile/water 50/50 (v/v) for other reagents (1 mM). The lipid solutions were irradiated 16 s for acetone, 50 s for benzaldehyde, 60 s for acetophenone, 20 s for benzophenone, 30 s for 2-acetylpyridine, 3-acetylpyridine, and 4-acetylpyridine.

### Mass spectrometry

Mass spectrometry (MS) analysis was carried out on a 4500 QTRAP triple quadrupole/linear ion trap (LIT) hybrid mass spectrometer (Applied Biosystems/Sciex, Toronto, Canada). All MS^4^ experiments were performed on an LTQ linear ion trap mass spectrometer (Thermo Fisher Scientific, San Jose, CA, USA). Considering the complexity of lipid extracts from real biological samples, neutral loss scan (NLS) were utilized to speed up targeted analysis of GPs of the same subclass or containing the same fatty acyl chains. For instance, neutral loss of the ion at *m/z* 183 can be used target PCs. Neutral losses of 141 Da and 185 Da are characteristic of PEs and PSs, respectively. For QTRAP 4500, the instrument parameters used were as follows: nanoelectrospray ionization (nanoESI) voltage, +1200–1800 V; curtain gas, 10 psi; interface heater temperature, 50 °C; declustering potential: 100 V; precursor ion isolation width was set to 1 Th; the collision energy (CE) used for MS^2^ experiments was 35–38 (arbitrary units, a.u.); the excitation energy (AF2) used for MS^3^ experiments was 0.1–0.14 a.u.; for MS^3^ experiments, ion injection time of 100–200 ms; for NLS, CE of 38–43 V; data analysis was done by the Analyst software for QTRAP 4500. For Thermo LTQ, the instrument parameters used were as follows: nanoESI voltage, +1500–1800 V; capillary temperature, 250 °C; capillary voltage, −10 V; tube lens voltage, 50 V; maximum injection time, 200 ms; microscans, 3; isolation width, 2 Th; normalized collision energy (NCE) settings of MS^2^, 38–40 a.u.; NCE settings of MS^3^, 27–29 a.u.; NCE settings of MS^4^, 24–26 a.u.

### Data analysis

The peak heights of the corresponding C=C and *sn*-isomers diagnostic ions of the target lipid species were extracted and analyzed throughout this study for quantitation. When performing statistical analyses of different lipid isomers, all isomer-specific ions were used to represent the amount of a certain isomer. Statistical analysis was partially carried out using Microsoft Excel 2016. For unsupervised multivariate data analysis, principal component analysis (PCA) and hierarchical cluster analysis were used. PCA was performed using an in-house Matlab routine on Matlab R2014b. HemI^67^ software (version 1.0.3.7) was used for hierarchical cluster analysis and visualization. Pearson's correlation coefficient was used for distance measurements and average linkage (default) variance was used as the clustering method.

### Reporting summary

Further information on research design is available in the Nature Research Reporting Summary linked to this article.

## Supplementary information

Supplementary Information

Reporting Summary

## Data Availability

All data supporting the findings of this study are available from the corresponding authors upon reasonable request. The source data for Figs. 1e, 2c, 4b–d, 5c and Supplementary Figs. 18, 19, 24, 26, 27, 29–32, and Supplementary Table 4 are provided in a Source Data file.

## Source data

Source Data
