# Supplementary material for: Large-scale lipid analysis with C=C location and sn-position isomer resolving power
Source: Nat Commun. 2020 Jan 17;11:375. doi: 10.1038/s41467-019-14180-4 (PMC6969141; doi:10.1038/s41467-019-14180-4)
Supplement: Supplementary file 1 — Supplementary Information [file 41467_2019_14180_MOESM1_ESM.pdf]

Supplementary Information

Large-scale lipid analysis with C=C location and *sn*-position isomer resolving power

Cao et al.

Contents:

Supplementary Figures 1-35
Supplementary Tables 1-12
Supplementary Note 1-3

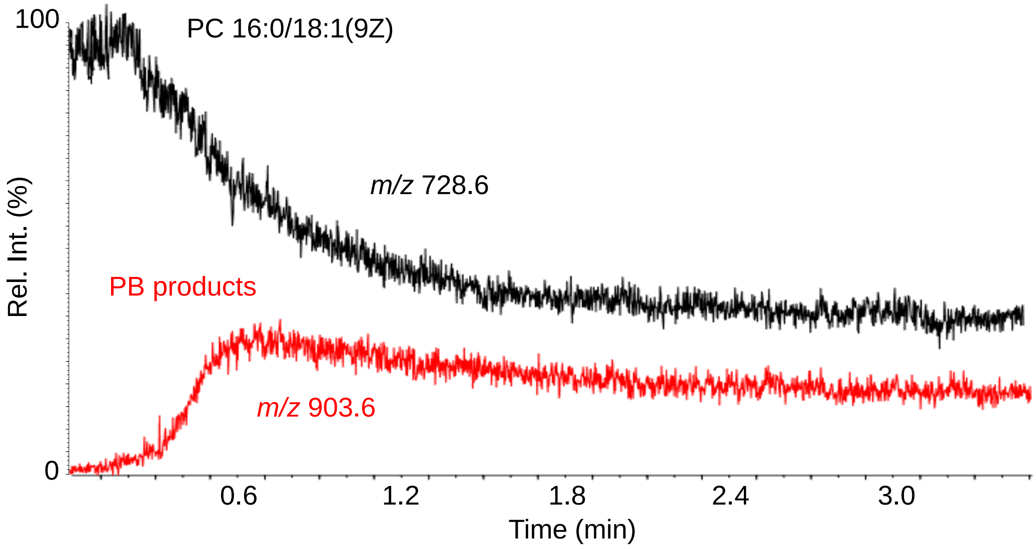

Supplementary Figure 1. PB reaction kinetics between 1 mM 2-acetylpyridine and 10 μM PC 16:0/18:1(9Z) (labelled in black), monitored by nanoESI-MS. The intensity of quasimolecular ions for the PB products (labelled in red) reached the maximum after ~30 seconds.

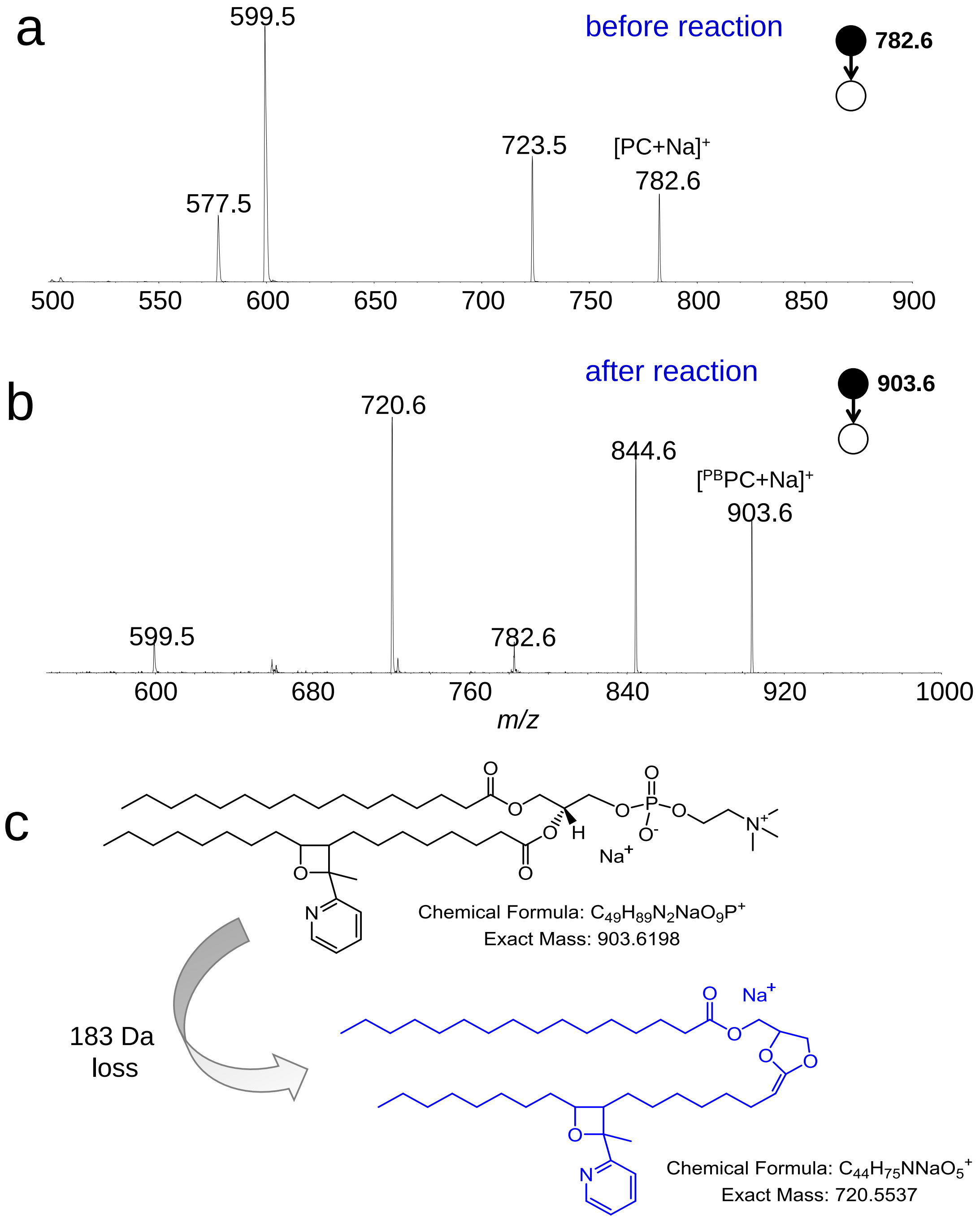

Supplementary Figure 2. MS/MS spectra of PC16:0/18:1(9Z) before (a) and (b) after PB reaction with 2-acetylpyridine. The predominant fragmentation channels were 59 Da and 183 Da losses, corresponding to losses of $N(CH_3)_3$ and PC headgroup. (c) Proposed structures of the PB product before and after headgroup loss (-183 Da) (labelled in blue).

Structures of C=C-specific diagnostic ions for PC 16:0/18:1(9Z)

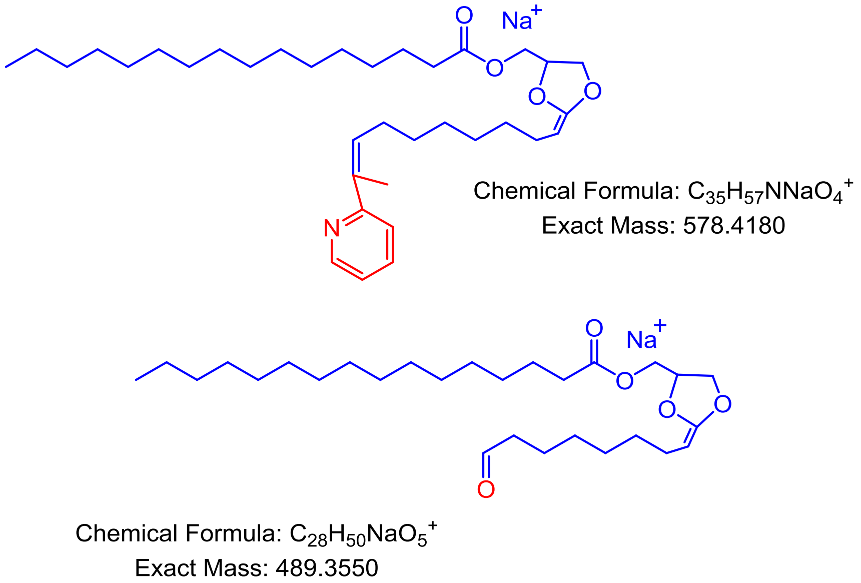

Supplementary Figure 3. Proposed structures of C=C-specific diagnostic ions for PC 16:0/18:1(9Z).

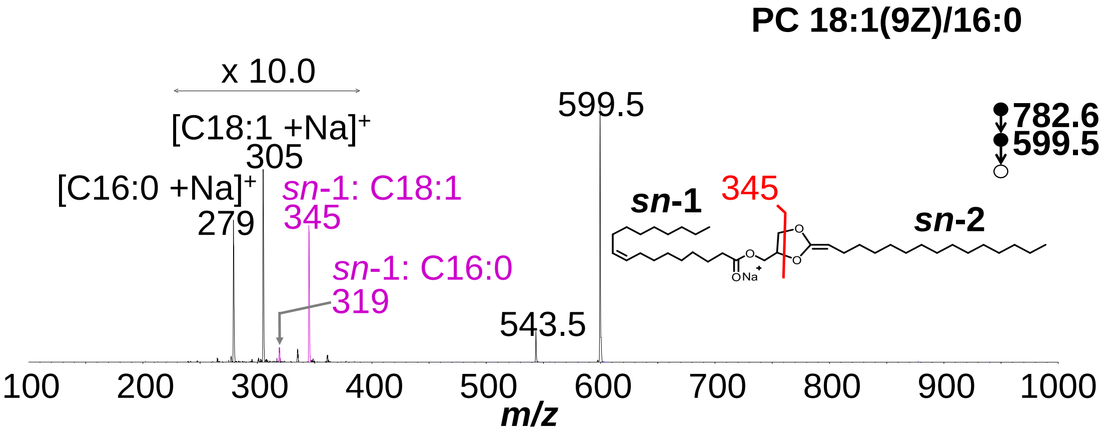

Supplementary Figure 4. MS^3 spectrum of PC 18:1(9Z)/16:0 without PB derivatization. Sodiated lipid precursors were first fragmented to generate products ion after headgroup loss (-183 Da). Products ion at *m/z* 599.5 were further fragmented to release *sn*-1-specific diagnostic ions (labelled in purple) at *m/z* 345 (C18:1) and 319 (C16:0).

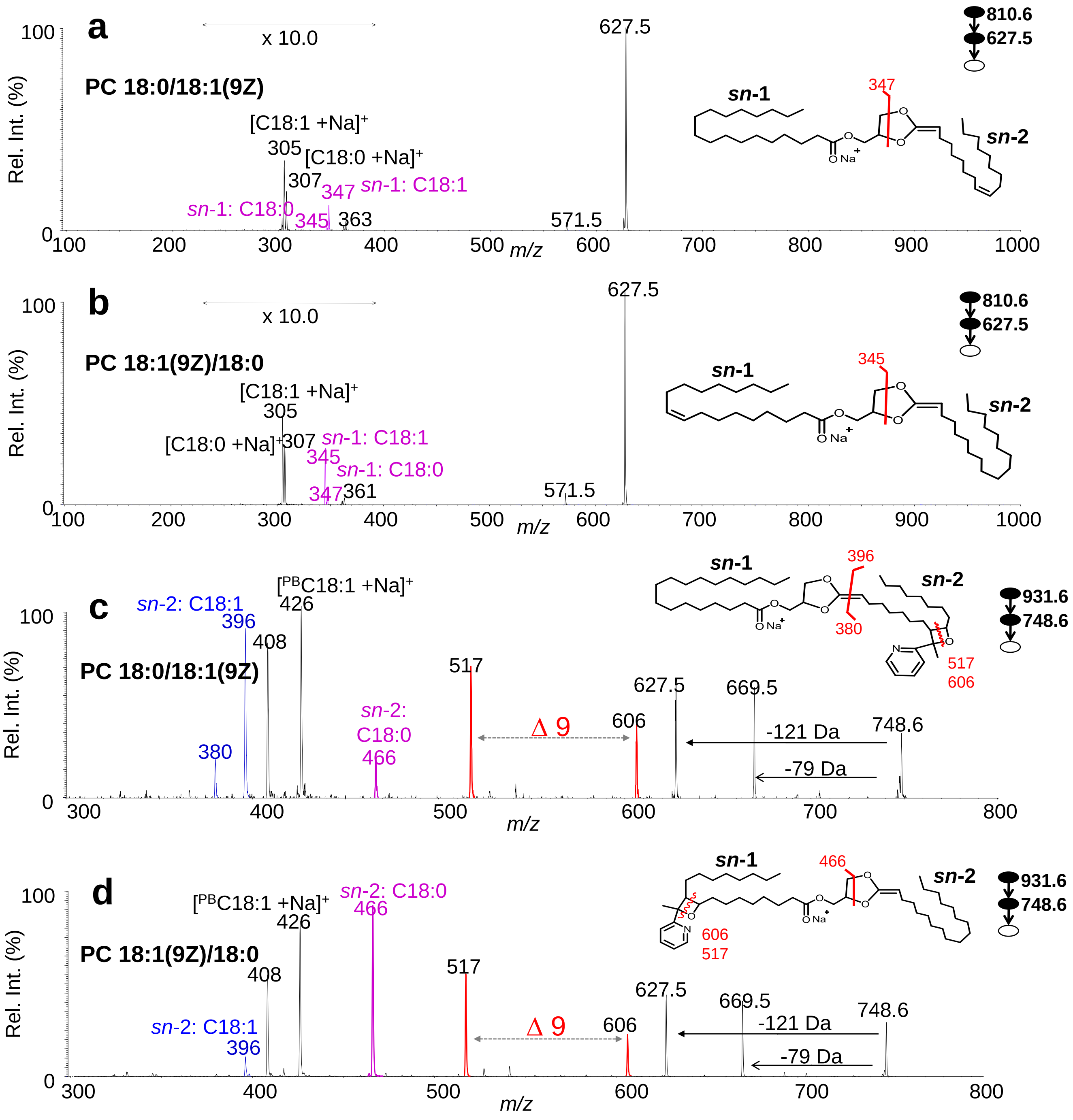

Supplementary Figure 5. MS^3 spectrum of PC 18:1(9Z)/16:0 without PB derivatization. (a, b) MS^3 spectra of sodiated intact PC 18:0/18:1(9Z) and PC 18:1(9Z)/18:0. (c, d) PB-MS^3 spectra of PC 18:0/18:1(9Z) and PC 18:1(9Z)/18:0 using 2-acetylpyridine. Note the significantly increased intensity of *sn*-specific ions (*m/z* 380, 396, and 466). (Peaks labelled in red are C=C-specific diagnostic ions, while peaks labelled in blue/purple are *sn*-specific diagnostic ions.)

Supplementary Table 1. Comparison of the absolute intensities of diagnostic ions without and with PB derivatization of PC 16:0_18:1(9Z) using 2-acetylpyridine.

Lipid	Intensity of C=C diagnostic ions (a.u.)		Intensity of *sn* diagnostic ions (a.u.)	
			***m/z* 319**	***m/z* 345**
PC 16:0/18:1(9Z)	/	/	84635	15931
PC 18:1(9Z)/16:0	/	/	9259	101851
	**Intensity of C=C diagnostic ions (a.u.)**		**Intensity of *sn* diagnostic ions (a.u.)**	
	m/z 489	*m/z* 578	m/z (396+380)	*m/z* 466
PBPC 16:0/18:1(9Z)	1173734	412456	1569694	267960
PBPC 18:1(9Z)/16:0	1211626	282170	172092	1996123
	**Signal enhancement**		**Signal enhancement**	
**PC 16:0_18:1(9Z)**	**∞**		**19 folds**	

Supplementary Table 2. Comparison of the absolute intensities of diagnostic ions without and with PB derivatization of PC 18:0_18:1(9Z) using 2-acetylpyridine.

	Intensity of C=C diagnostic ions (a.u.)		Intensity of *sn* diagnostic ions (a.u.)	
			**m/z 345**	**m/z 347**
PC 18:0/18:1(9Z)	/	/	10888	72987
PC 18:1(9Z)/18:0	/	/	89147	10296
	**Intensity of C=C diagnostic ions (a.u.)**		**Intensity of *sn* diagnostic ions (a.u.)**	
	***m/z* 517**	***m/z* 606**	***m/z* (396+380)**	***m/z* 466**
PBPC 18:0/18:1(9Z)	965512	567048	1432944	186512
PBPC 18:1(9Z)/18:0	723911	269719	159445	1654372
	**Signal enhancement**		**Signal enhancement**	
**PC 18:0_18:1(9Z)**	**∞**		**20 folds**	

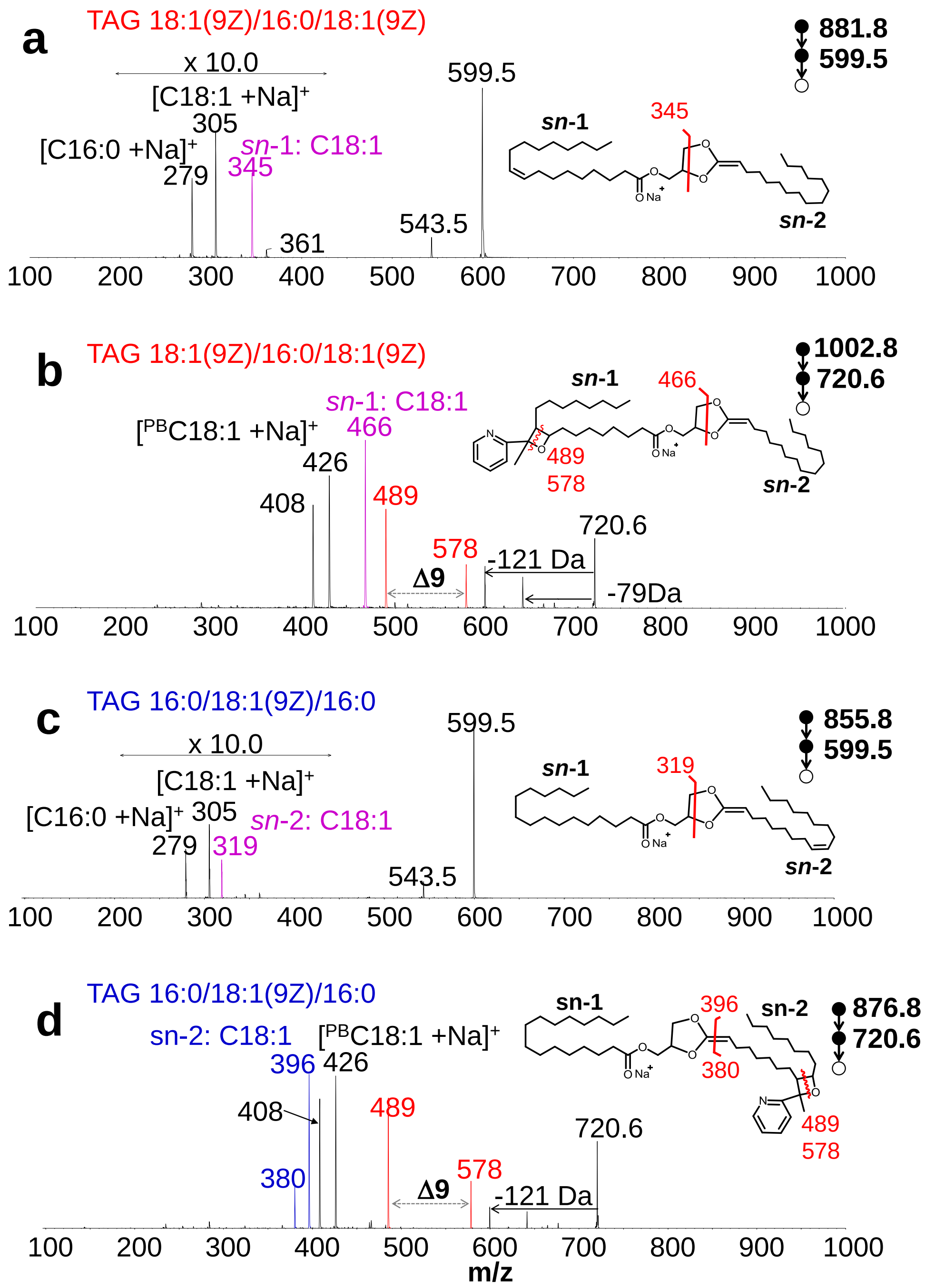

Supplementary Figure 6. Use of TAG 18:1(9Z)/16:0/18:1(9Z) and TAG 16:0/18:1(9Z)/16:0 for method validation. (a) MS^3 spectrum of TAG 18:1/16:0/18:1 after loss of one C18:1 chain via CID. (b) PB-MS^3 spectrum of TAG 18:1/16:0/18:1 after loss of one C18:1 chain via CID. (c) MS^3 spectrum of TAG 16:0/18:1/16:0 after loss of one C16:0 chain via CID. (d) PB-MS^3 spectrum of TAG 16:0/18:1/16:0 after loss of one C16:0 chain via CID. (Peaks labelled in red are C=C-specific diagnostic ions, while peaks labelled in blue/purple are *sn*-specific diagnostic ions.)

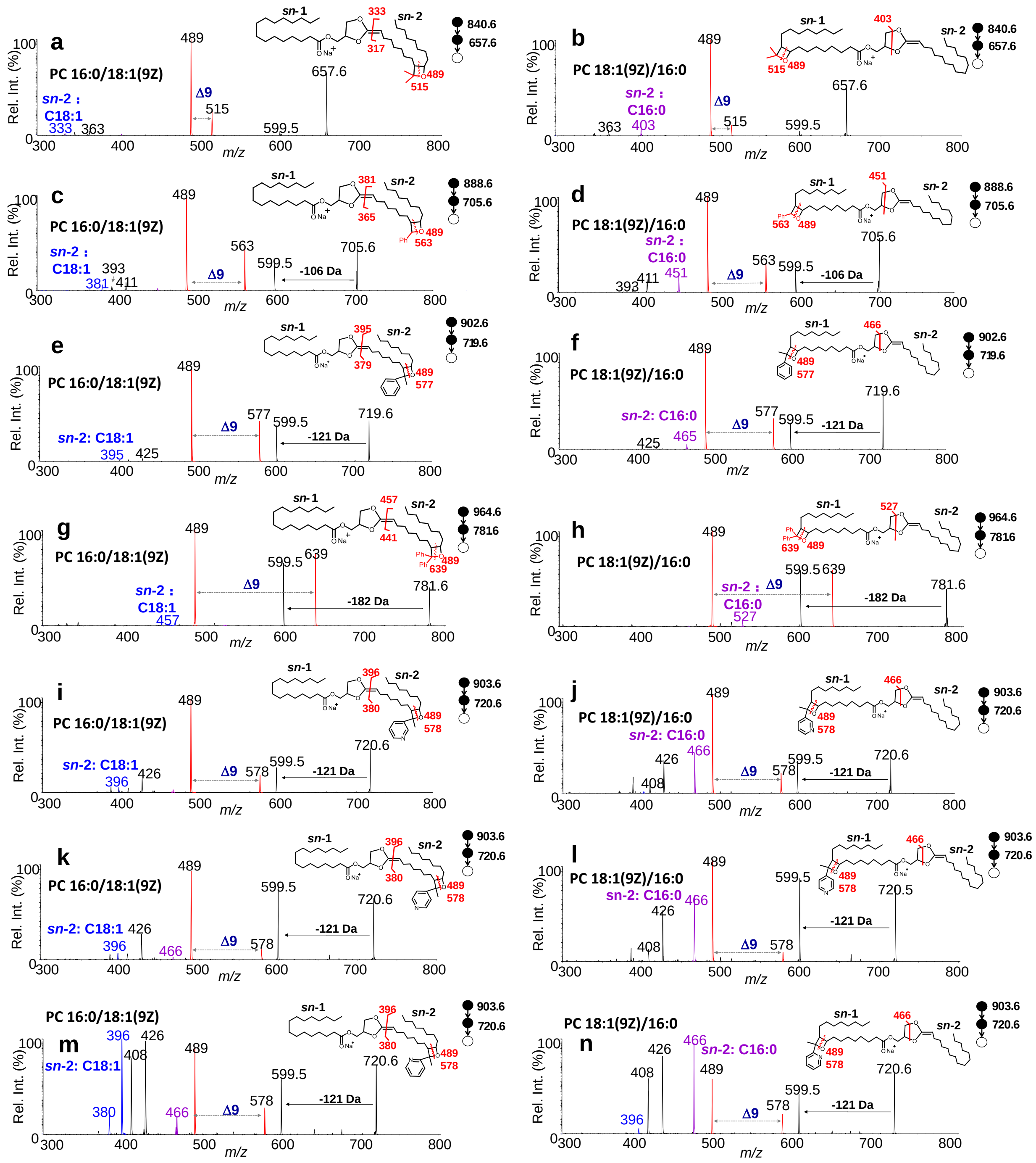

Supplementary Figure 7. PB-MS^3 analysis of PC 16:0_18:1(9Z) *sn*-isomers using different PB reagents. PB-MS^3 spectra of PC 16:0/18:1(9Z) and PC 18:1(9Z)/16:0 using (a, b) acetone, (c, d) benzaldehyde, (e, f) acetophenone, (g, h) benzophenone, (i, j) 3-acetylpyridine, (k, l) 4-acetylpyridine, and (m, n) 2-acetylpyridine, respectively. Highly abundant C=C-specific diagnostic ions (labelled in red) were produced for each PC *sn*-isomer for all PB reagents tested. The efficiency of producing *sn*-specific ions (labelled in blue/purple) were varied for different PB reagents, among which 2-acetylpyridine worked the best.

Supplementary Table 3. List of carbonyl compounds tested as PB reagents for the PB-MS^3 workflow.

**Aldehyde/ketone**	**Mass increase of PB products (Da)**	**Structure**	**Mass difference between C=C diagnostic ions (Da)**	**PB reaction and PB products fragmentation features**
Acetone, liquid at room temperature	58	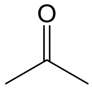	26	■ Low mass increase in PB products;<br>■ High reaction kinetics (15~20 s);<br>■ Miscible with common ESI solvents and compatible with (nano)ESI;<br>■ Side reactions;<br>■ C=C-ions: high abundance;<br>■ *sn*-ions: low abundance.
Benzaldehyde, liquid at room temperature	106	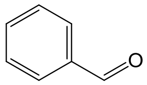	74	■ Adequate mass increase in PB products;<br>■ Adequate reaction kinetics (~1 min);<br>■ Amount in ESI solvents is limited due to high viscosity and solubility issues;<br>■ Less side reactions;<br>■ C=C-ions: high abundance;<br>■ *sn*-ions: low abundance.
Acetophenone, liquid at room temperature	120	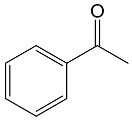	88	■ High mass increase in PB products;<br>■ Adequate reaction kinetics (~1 min);<br>■ Less side reactions;<br>■ C=C-ions: high abundance;<br>■ *sn*-ions: low abundance.
Benzophenone, solid at room temperature	182	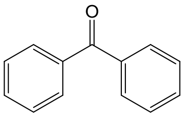	150	■ High mass increase in PB products;<br>■ High reaction kinetics (20~30 s);<br>■ Less side reaction;<br>■ C=C-ions: high abundance;<br>■ *sn*-ions: low abundance.
2-acetylpiridine, liquid at room temperature	121	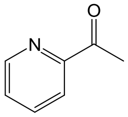	89	■ High mass increase in PB products;<br>■ Fast reaction kinetics (~30 s);<br>■ Negligible side reactions;<br>■ C=C-ions: high abundance;<br>■ *sn*-ions: high abundance.
3-acetylpiridine, liquid at room temperature	121	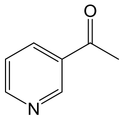	89	■ High mass increase in PB products;<br>■ Fast reaction kinetics (~30 s);<br>■ Negligible side reactions;<br>■ C=C-ions: high abundance;<br>■ *sn*-ions: adequate abundance.
4-acetylpiridine, liquid at room temperature	121	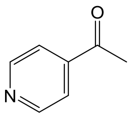	89	■ High mass increase in PB products;<br>■ Fast reaction kinetics (~30 s);<br>■ Negligible side reactions;<br>■ C=C-ions: high abundance;<br>■ *sn*-ions: adequate abundance.

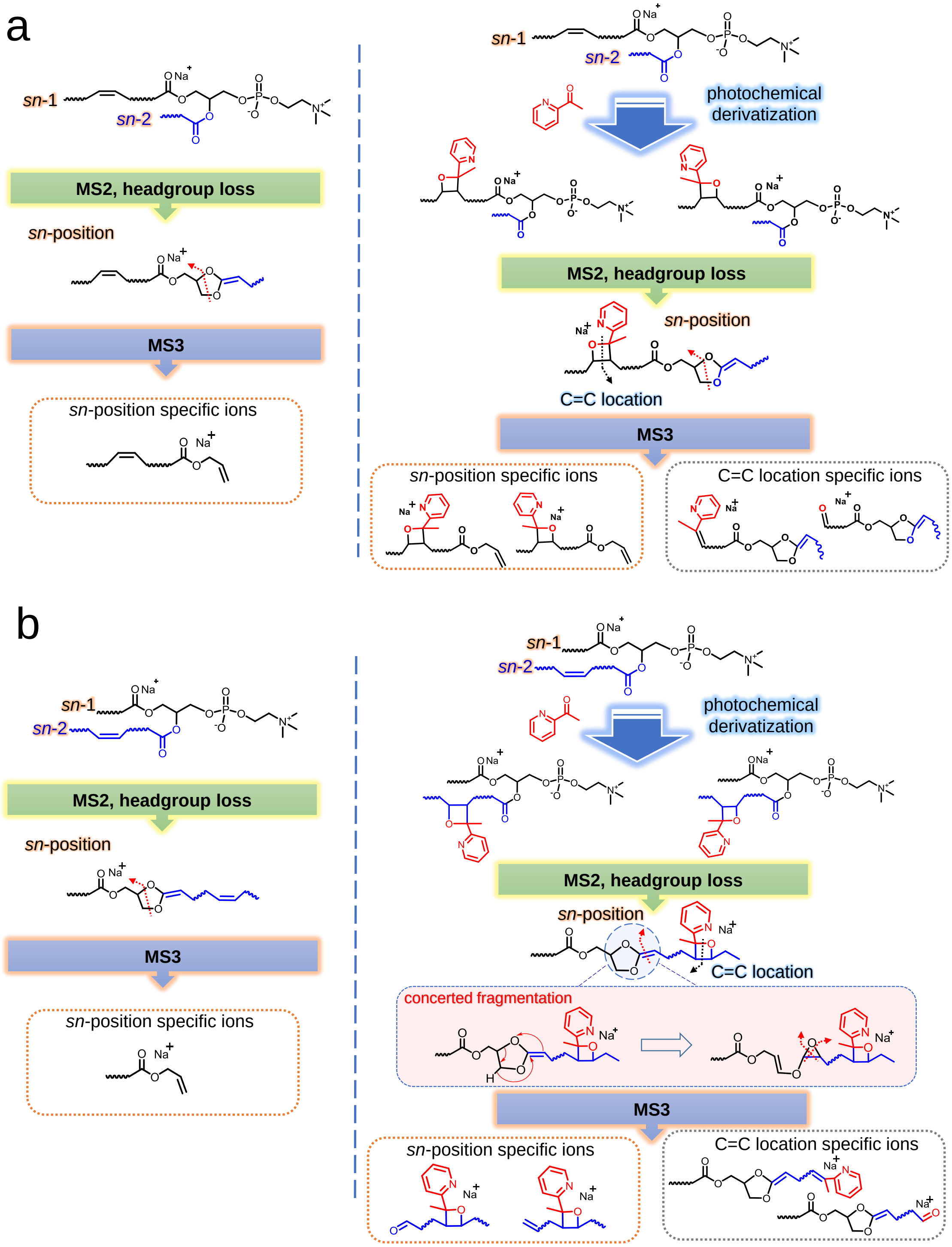

Supplementary Figure 8. Proposed mechanisms for the production of C=C- and *sn*-specific diagnostic ions in this study. (a) C=C at *sn*-1. (b) C=C at *sn*-2.

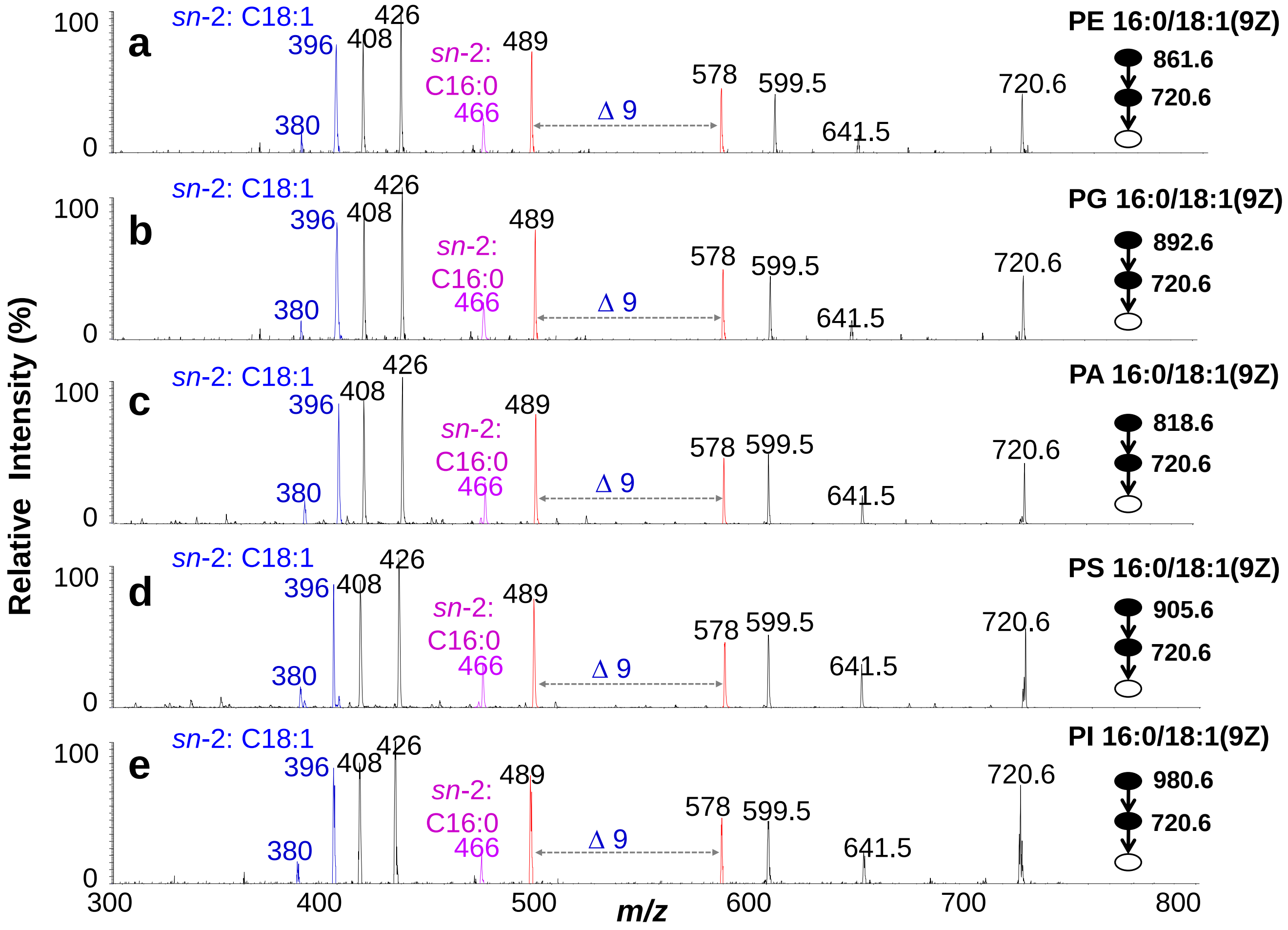

Supplementary Figure 9. The PB-MS^3 method can be applied for the analysis of various types of GPs, including PC, PE, PG, PA, PS, and PI. PB-MS^3 spectra of (a) PE 16:0/18:1(9Z), (b) PG 16:0/18:1(9Z), (c) PA 16:0/18:1(9Z), (d) PS 16:0/18:1(9Z), and (e) PI 16:0/18:1(9Z). (Peaks labelled in red are C=C-specific diagnostic ions, while peaks labelled in blue/purple are *sn*-specific diagnostic ions.)

Supplementary Table 4. Comparison of the percentage of regioisomeric impurity of synthetic lipid standards using PLA_2 assay and the $PB\text{-}MS^3$ method. Source data is provided in a Source Data file.

Lipid Standard	PLA_2 assay (%)	$PB\text{-}MS^3$ (%)
PC 16:0/18:1	16.4 ± 1.9	14.8 ± 1.4
PC 18:1/16:0	8.1 ± 1.6	7.5 ± 0.8
PC 18:0/20:4	11.7 ± 2.5	10.3 ± 1.1
PE 16:0/18:1	21.5 ± 3.7	23.4 ± 2.2
PS 16:0/18:1	22.8 ± 3.9	24.1 ± 1.9
PG 16:0/18:1	21.6 ± 3.6	23.4 ± 2.0
PA 16:0/18:1	23.5 ± 4.4	21.9 ± 2.2
PI 16:0/18:1	18.7 ± 4.2	20.3 ± 1.8

Supplementary Table 5. The formation efficiency of the headgroup-loss MS^2 fragment and the limit of detection (LOD) for each lipid class.

Lipid class	Lipid standard	Headgroup loss transition	Limit of detection (LOD)
Phosphatidylcholine	PC 16:0/18:1	52% (903 → 720)	5 nM
Phosphatidylethanolamine	PE 16:0/18:1	41% (861 → 720)	10 nM
Phosphatidylserine	PS 16:0/18:1	37% (905 → 720)	40 nM
Phosphatidylglycerol	PG 16:0/18:1	40% (892 → 720)	35 nM
Phosphatidic	PA 16:0/18:1	42% (818 → 720)	50 nM
Phosphatidylinositol	PI 16:0/18:1	29% (980 → 720)	50 nM

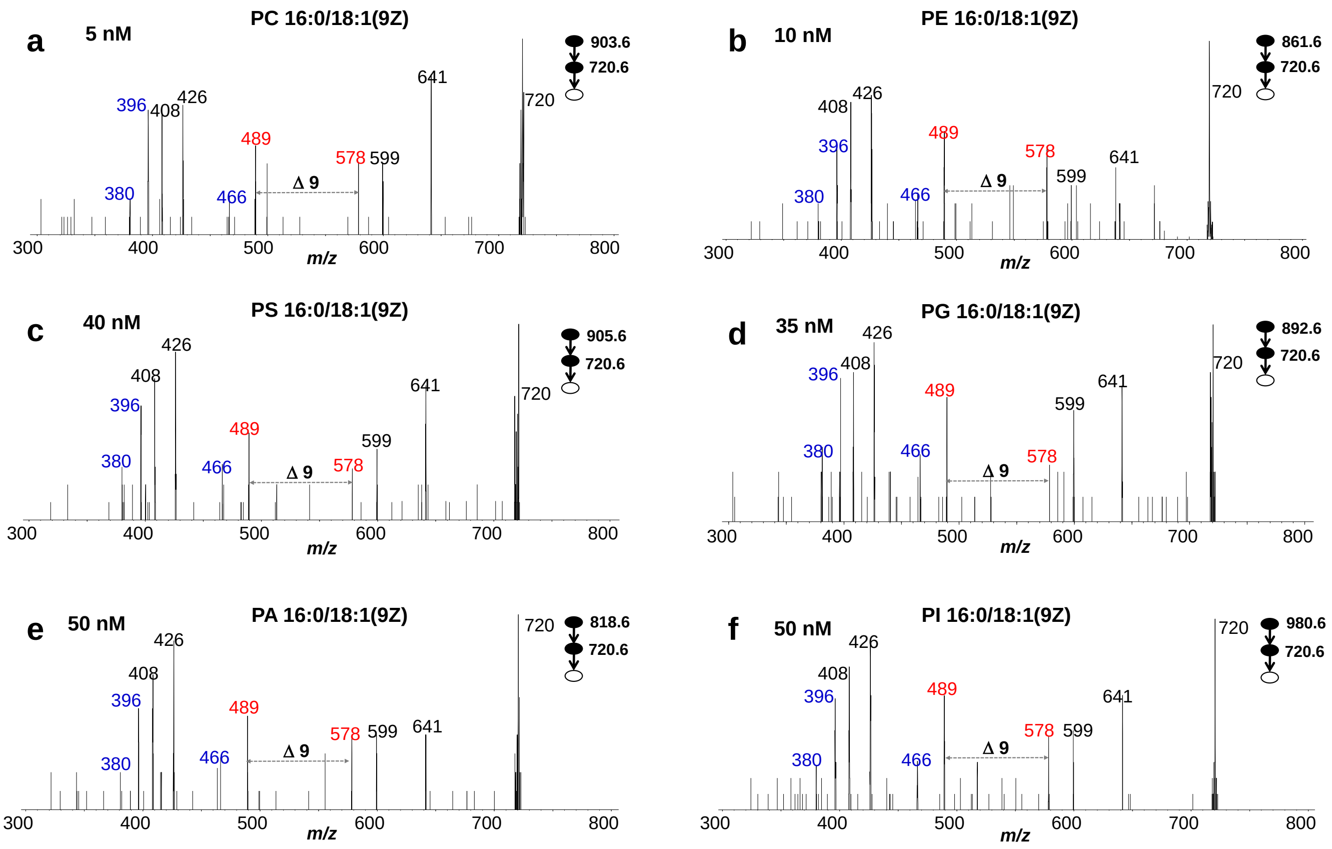

Supplementary Figure 10. Estimation of LODs via a S/N of ~3 for diagnostic ions in PB-MS^3 spectra**.** PB-MS^3 spectra of (a) PC 16:0/18:1(9Z) (5 nM), (b) PE 16:0/18:1(9Z) (10 nM), (c) PS 16:0/18:1(9Z) (40 nM), (d) PG 16:0/18:1(9Z) (35 nM), (e) PA 16:0/18:1(9Z) (50 nM), and (f) PI 16:0/18:1(9Z) (50 nM). (Peaks labelled in red are C=C-specific diagnostic ions, while peaks labelled in blue/purple are *sn*-specific diagnostic ions.) Since the high affinity of the pyridine towards sodium/proton, leading to high ionization efficiency of the PB products of lipids derivatized using 2-acetylpyridine.

Supplementary Note 1. PB-MS^3 coupled with LC-MS.

We demonstrated that our workflow is in principle compatible with the LC-MS^n workflow by analyzing a pair of lipid *sn*-isomers. Two PC standards (PC 16:0/18:1(9Z) and PC 18:1(9Z)/16:0) were constituted as a mixture with the same molar concentration. The base peak chromatogram (BPC) for sodiated PB products of PC (^{PB}PC 34:1) at *m/z* 903 (Supplementary Fig. 11a) was a single broad peak at the retention time (RT) of 11.8 min, with no resolution between the two *sn*-isomers. However, when the PB-MS^3 mass spectra are averaged across data points from an early eluting portion of the chromatographic peak (RT 11.2-11.6 min) an abundant ion of *m/z* 466 consistent with a C18:1 at *sn*-1 position was observed, along with a less abundant ion of *m/z* 396 arising from a C18:1 at *sn*-2 (Supplementary Fig. 11c). When the PB-MS^3 mass spectra are averaged across data points from a later eluting portion of the chromatographic peak (RT 12.1−12.5 min), the MS signal at *m/z* 396 became more abundant, which was consistent with a C18:1 at *sn*-2 position (Supplementary Fig. 11d). In this way the two *sn*-isomers were partially separated, with PC 18:1(9Z)/16:0 eluting slightly earlier than PC 16:0/18:1(9Z) (Supplementary Fig. 11b). The diagnostic ions of C=C location were also observed in the LC-MS^n workflow (Supplementary Fig. 11c,d). This experiment has clearly demonstrated that our method was compatible with LC-MS^n.

Experimental: LC-MS^n analyses were performed on an ExionLC AC system coupled to a 4500 QTRAP mass spectrometer (Sciex). A mixture of lipid standards (10 μM, 2 μL) after PB reaction were injected into a HILIC column (150 mm × 2.1 mm, silica spheres, 2.7μm, Sigma-Aldrich) held at 30 ℃ at a flow rate of 200 μL/min flow rate. The mobile phase consisted of A: acetone/ACN (50/50,v/v) and B: ammonium acetate aqueous solution (10 mM). Gradient elution was applied for separation (A started from 90%, decreased to 85% at 5 min, then decreased to 80% at 8 min, then 80% during 8–15 min, and decreased to 70% in 16 min until after 20 min). Sodium acetate (20 mM) was infused into the flow postcolumn using a tee-union at a flow rate of 1 μL/min to promote sodium adduct formation. MS parameters were as follows: ESI voltage (+4500 V), curtain gas (35 psi), interface heater temperature (400 ℃), nebulizing gas (GS1, 30 psi), and nebulizing gas (GS2, 30 psi). Precursor ion isolation width was set to 1 Th. A collision energy (CE) of 38 a.u. was used for MS^2 experiments. The excitation energy (AF2) used for MS3 experiments was 0.13 a.u. For MS^3 experiments, the ion injection time was 200 ms.

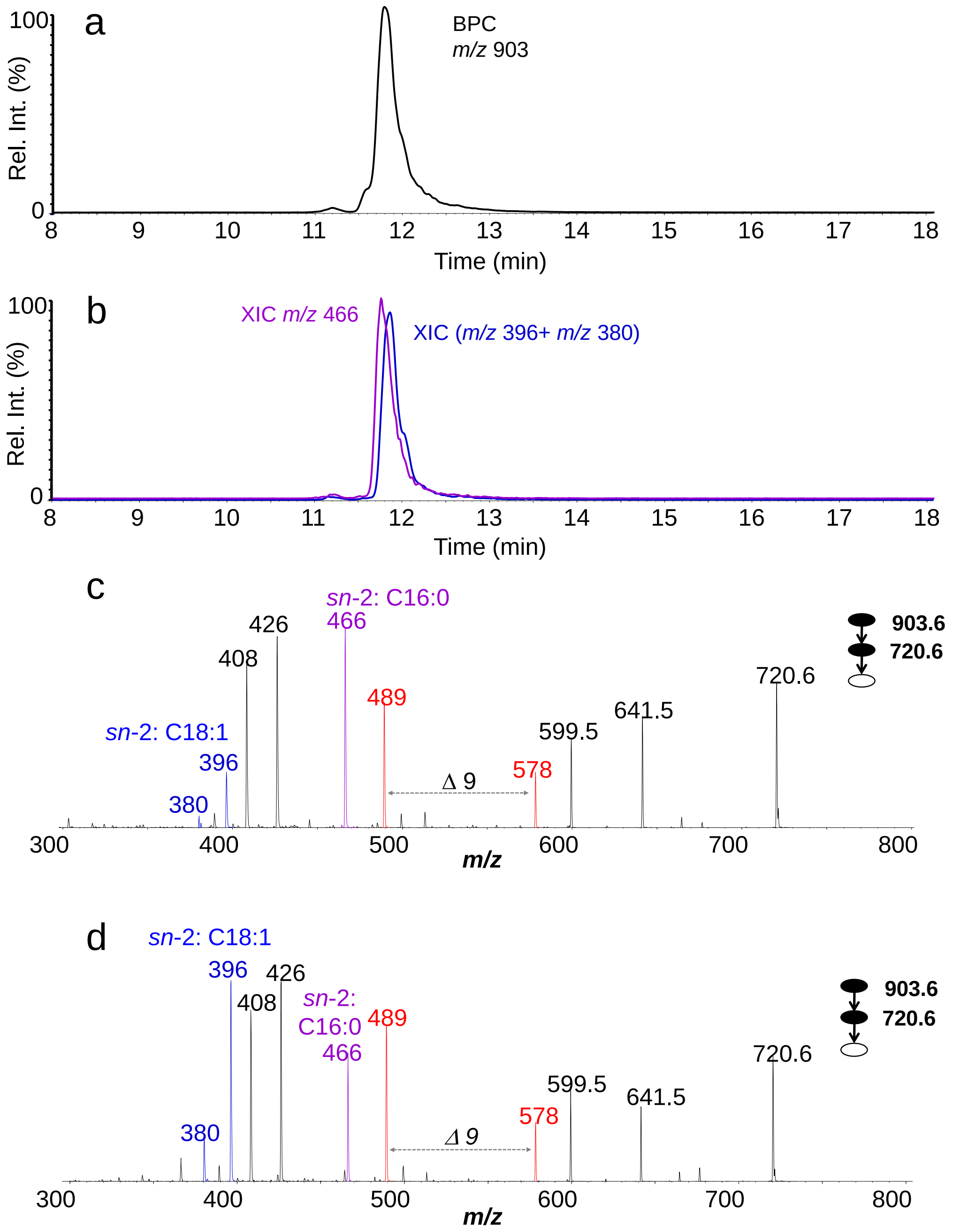

Supplementary Figure 11. LC-MS analysis of two lipid isomer standards (PC 16:0/18:1(9Z) and PC 18:1(9Z)/16:0) mixed in the same molar concentration. (a) The base peak chromatogram (BPC) for sodiated PB products of PC (^{PB}PC 34:1) at *m/z* 903. (b) The extracted ion chromatogram (XIC) labelled in purple of *sn*-specific ions of *m/z* 466, indicative of PC 18:1(9Z)/16:0, while the XIC labelled in blue of *m/z* 396/380, indicative of PC 16:0/18:1(9Z). (c) The PB-MS^3 mass spectrum was averaged across data points from an early eluting portion of the chromatographic peak (RT 11.2-11.6 min). (d) The PB-MS^3 mass spectrum was averaged across data points from a later eluting portion of the chromatographic peak (RT 12.1−12.5 min). (Peaks labelled in red are C=C-specific diagnostic ions, while peaks labelled in blue/purple are *sn*-specific diagnostic ions.)

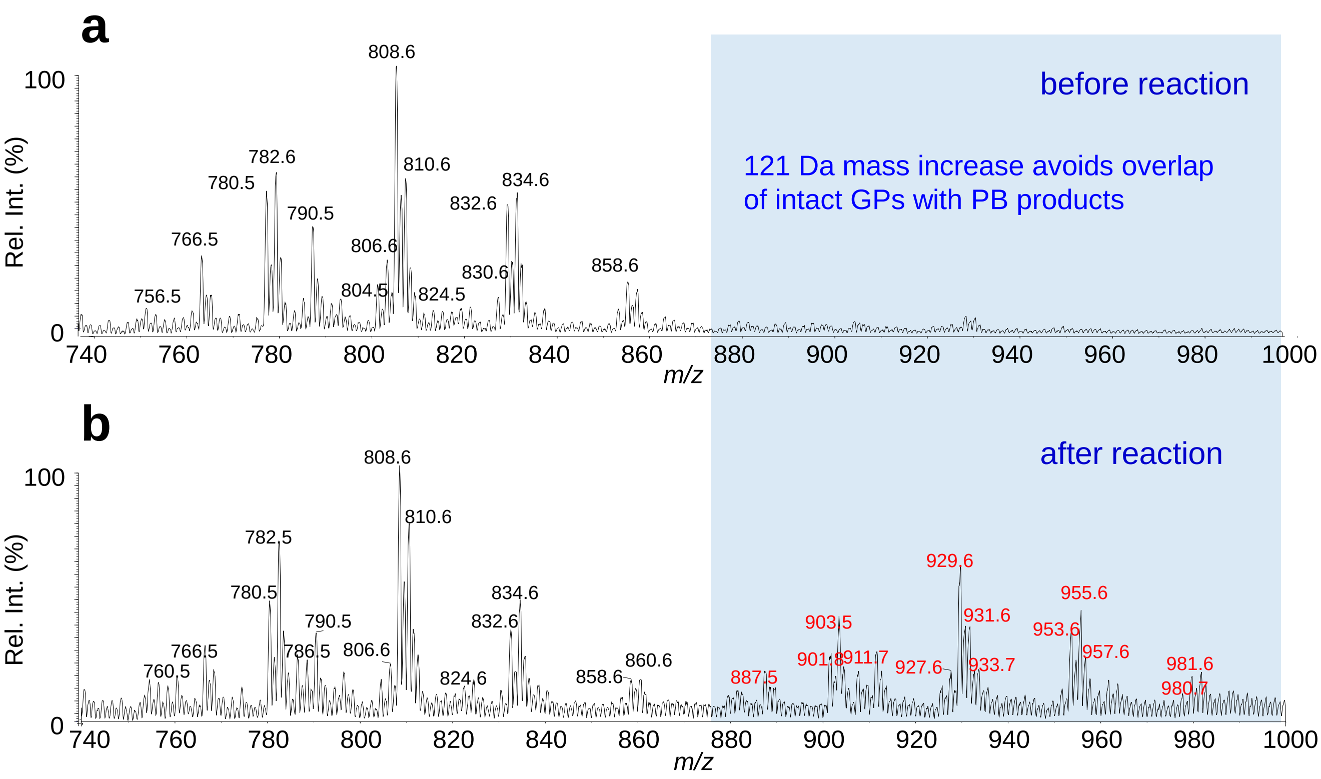

Supplementary Figure 12. NanoESI spectra of the bovine liver polar extract before (a) and after (b) PB reaction. Note that the cluster of quasimolecular ions for PB products (shaded in blue) do not overlap with intact lipid peaks.

Relative amount of PC 16:0/20:3(8,11,14) in PC 36:3 in bovine liver polar extract

$$\frac{\text{intensity of } sn\text{−ions for isomer 1}}{\text{intensity of } sn\text{−ions for all PC 36:3 isomers}} = \frac{I_{420}}{I_{420}+I_{392}+I_{466}+I_{394}+I_{464}+I_{396}}$$

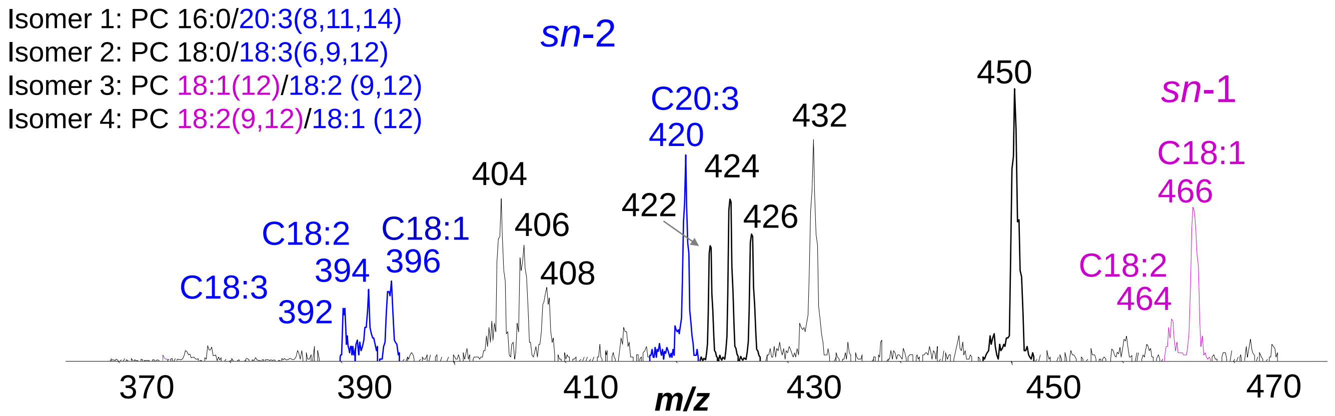

Supplementary Figure 13. Use of the *sn*-specific ions for the relative quantitation of *sn*-position isomers of GPs, using PC 36:3 in the bovine polar lipid extract as an example. (Peaks labelled in blue/purple are *sn*-specific diagnostic ions.)

Supplementary Table 6. Identified GPs from bovine liver polar extract by PB-MS^3. Both C=C and *sn*-positions were determined for each lipid species (blue: *sn*-positional isomers, red: C=C location isomers).

PLs	Precursor $[M+Na]^+$ (*m/z*)	Headgroup loss ion (*m/z*)	Diagnostic ions for *sn*-position (*m/z*)	Diagnostic ions for double bond position (m/z)
PC 16:0/16:0	756.5	573.5	319	
PC 16:0/18:2(9, 12)	901.6	718.6	378, 394	489,578; 529, 618
PC 18:2(9, 12)/16:0	901.6	718.6	464	489,578; 529, 618
PC 16:0/18:1(9)	903.6	720.6	380, 396	489, 578
PC 16:0/18:1(11)	903.6	720.6	380, 396	517, 606
PC 18:1(9)/16:0	903.6	720.6	466	489, 578
PC 18:1(11)/16:0	903.6	720.6	466	517, 606
PC 16:0/20:4(5, 8, 11, 14)	925.6	742.6	402, 418	433, 522; 473, 562; 513, 602; 553, 642
PC 20:4(5, 8, 11, 14)/16:0	925.6	742.6	488	433, 522; 473, 562; 513, 602; 553, 642
PC 16:0/20:3(8, 11, 14)	927.6	744.6	404, 420	475, 564; 515, 604; 555, 644
PC 18:0/18:3(6, 9, 12)	927.6	744.6	376, 392	475, 564; 515, 604; 555, 644
PC 18:1(12)/18:2(9, 12)	927.6	744.6	378, 394, 466	515, 604; 555, 644
PC 18:2(9,12)/18:1(12)	927.6	744.6	380, 396, 464	515, 604; 555, 644
PC 18:0/18:2(9, 12)	929.6	746.6	378, 394	515, 604; 555, 644
PC 18:2(9, 12)/18:0	929.6	746.6	464	517, 606; 557, 646
PC 18:0/18:1(9)	931.6	748.6	380, 396	517, 606
PC 18:0/18:1(11)	931.6	748.6	380, 396	545, 634
PC 18:1(9)/18:0	931.6	748.6	466	517, 606
PC 18:1(11)/18:0	931.6	748.6	466	545, 634
PC 18:0/20:4(5, 8, 11, 14)	953.6	770.6	402, 418	461, 550; 501, 590; 541, 630; 581, 670
PC 16:0/22:4(7, 10, 13, 16)	953.6	770.6	430, 446	461, 550; 501, 590; 541, 630; 581, 670
PC 18:0/20:3(8, 11, 14)	955.6	772.6	404, 420	503, 592; 543, 632; 583, 672
PC 18:0/22:5(4, 7, 10, 13, 16)	979.6	796.6	428, 444	447, 536; 487, 576; 527, 616; 567, 656; 607, 696
PC 18:0/22:4(7, 10, 13, 16)	981.6	798.6	430, 446	489, 578; 529, 618; 569, 658; 609, 698
PE 18:0/18:2 (9, 12)	887.6	746.6	378, 394	517, 606; 557, 646
PE 18:2(9, 12)/18:0	887.6	746.6	464	517, 606; 557, 646

PE 18:1(9)/18:1(9)	887.6	746.6	466, 380, 396	515, 604
PE 18:0/20:4(5,8,11,14)	911.6	770.6	402, 418	461, 550; 501, 590; 541, 630; 581, 670
PE 20:4(5,8,11,14)/18:0	911.6	770.6	488	461, 550; 501, 590; 541, 630; 581, 670
PE 16:0/22:4 (7, 10, 13, 16)	911.6	770.6	430, 446	461, 550; 501, 590; 541, 630; 581, 670
PE 18:0/20:3(8,11,14)	913.6	772.6	404, 420	503, 592; 543, 632; 583, 672
PE 20:3(8,11,14)/18:0	913.6	772.6	490	503, 592; 543, 632; 583, 672
PS 16:0/18:2 (9, 12)	903.6	718.6	378, 394	489,578; 529, 618
PS 18:2(9, 12)/16:0	903.6	718.6	464	489,578; 529, 618
PS 18:0/18:2 (9, 12)	931.6	746.6	378, 394	517, 606; 557, 646
PS 18:2(9, 12)/18:0	931.6	746.6	464	517, 606; 557, 646
PS 18:1(9)/18:1(9)	931.6	746.6	466; 380, 396	515, 604
PS 20:4(5, 8, 11, 14) /18:0	955.6	770.6	488	461, 550; 501, 590; 541, 630; 581, 670
PS 18:0/20:4 (5, 8, 11, 14)	955.6	770.6	402, 418	461, 550; 501, 590; 541, 630; 581, 670
PS 16:0/22:4 (7, 10, 13, 16)	955.6	770.6	430, 446	461, 550; 501, 590; 541, 630; 581, 670
PI 20:4(5, 8, 11, 14) /18:0	1030.6	770.6	488	461, 550; 501, 590; 541, 630; 581, 670
PI 18:0/20:4 (5, 8, 11, 14)	1030.6	770.6	402, 418	461, 550; 501, 590; 541, 630; 581, 670
PI 16:0/22:4 (7, 10, 13, 16)	1030.6	770.6	430, 446	461, 550; 501, 590; 541, 630; 581, 670
PI 20:3(8, 11, 14)/18:0	1032.6	772.6	490	503, 592; 543, 632; 583, 672
PI 18:0/20:3 (8, 11, 14)	1032.6	772.6	404, 420	503, 592; 543, 632; 583, 672

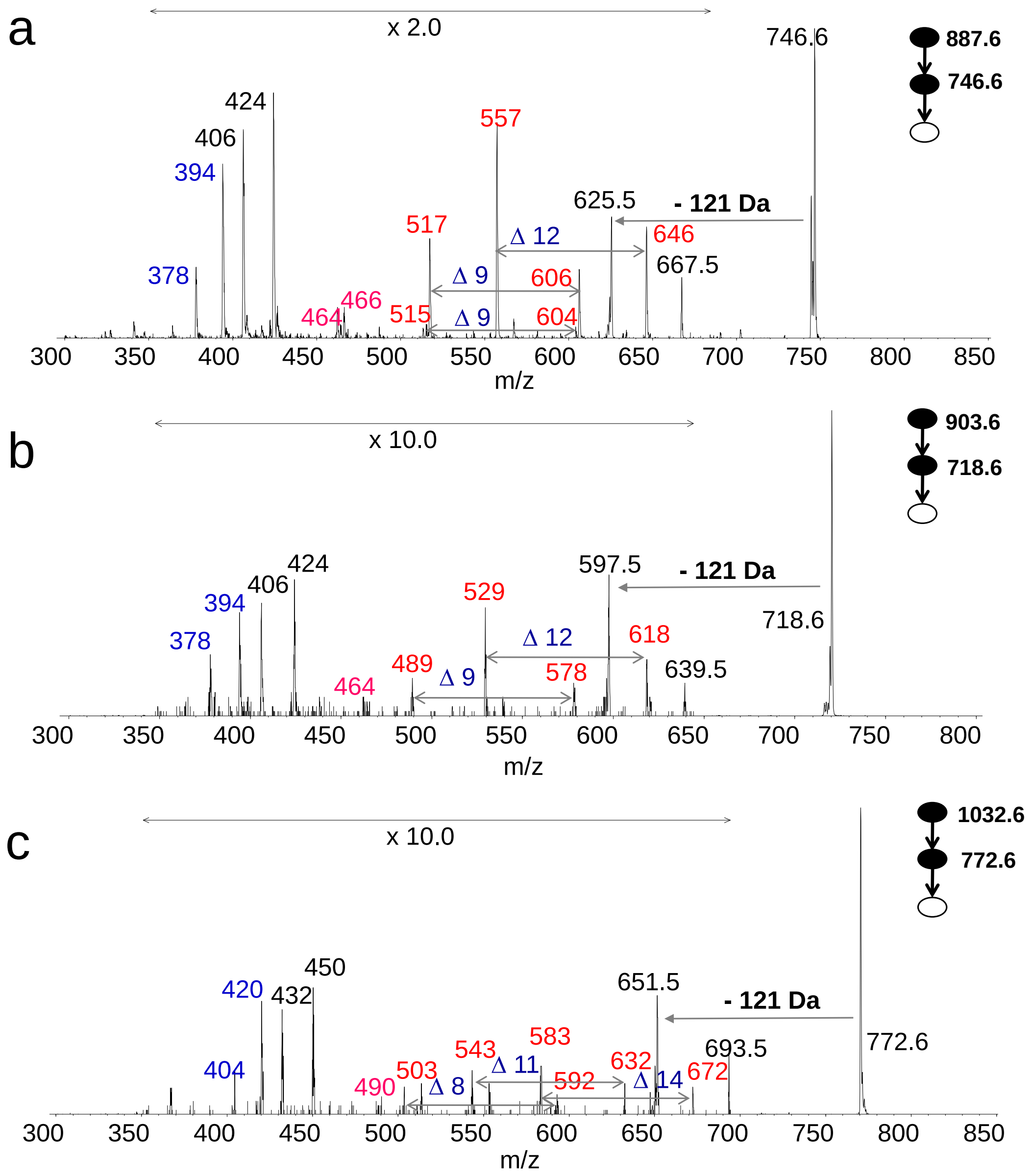

Supplementary Figure 14. Representative PB-MS^3 spectra of (a) PE(36:2), (b) PS(34:2) and (c) PI(38:3) from bovine liver polar extract. (Peaks labelled in red and blue/purple are C=C- and *sn*-specific diagnostic ions, respectively.)

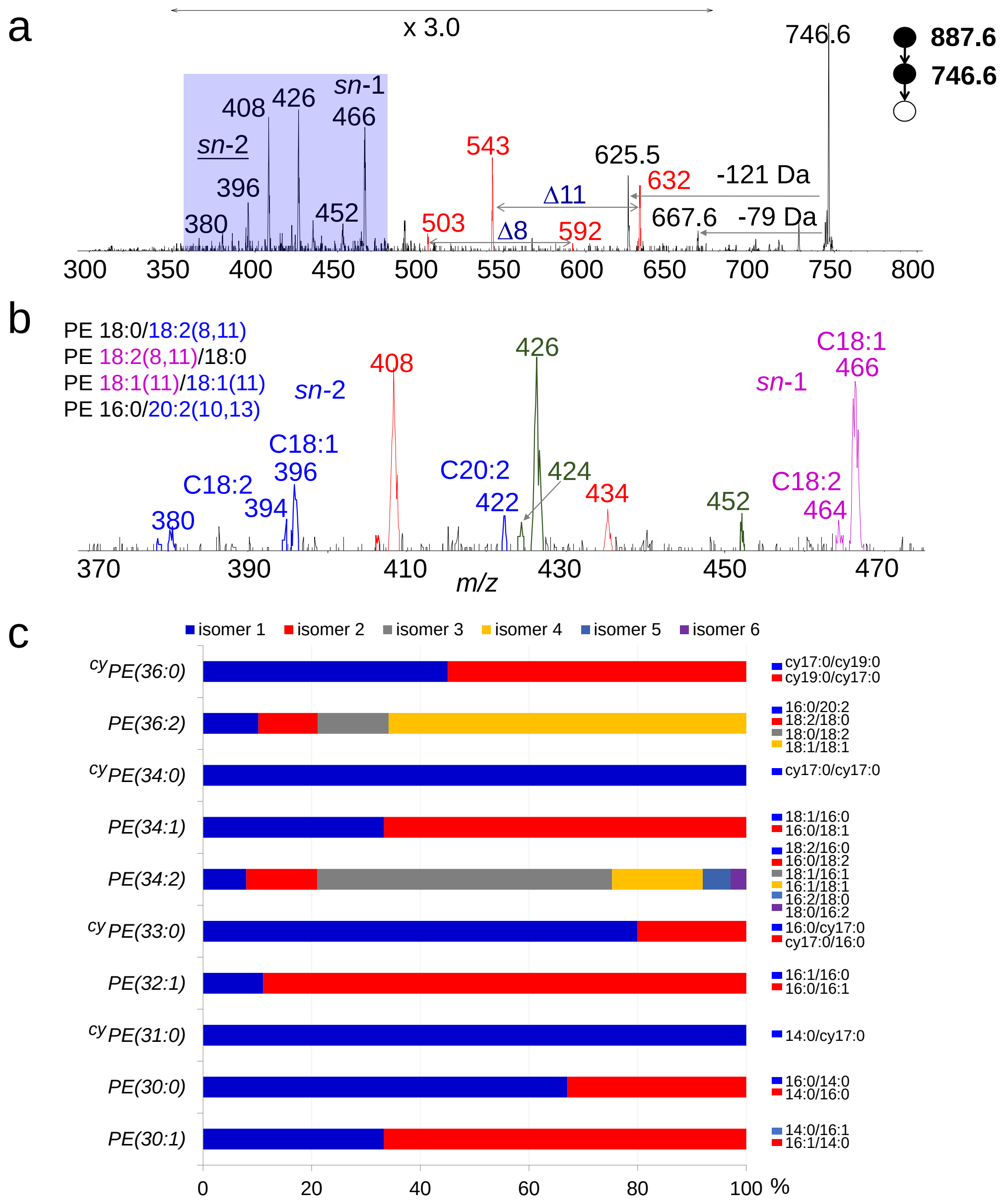

Supplementary Figure 15. Analysis of GP C=C and *sn*-position isomers in the lipid extract of E. Coli. (a) The MS^3 spectrum of PE 36:2 after loss of headgroup by CID. C=C-specific diagnostic ions were labelled in red, while ions reporting the *sn*-positions of fatty acyls were shaded in blue. (b) A magnified view of the shaded *m/z* region in (a) showing the set of fatty acyls at *sn*-2 position, including C18:1, C18:2, and C20:2(labelled in blue), the set of fatty acyls at *sn*-1 position including C18:1, and C18:2 (labelled in purple), sodiated PB products of FA 18:1, FA 18:2, and FA 20:2 at m/z 426, 424, and 452 (labelled in dark green) and the corresponding H_2O loss peaks (labelled in red). (c) The relative composition of *sn*-position isomers of GPs in E. coli extract. The relative amount was the intensity of the *sn*-specific diagnostic ions for a specific isomer divided by the total intensity of those for all possible *sn*-isomers (*cy*: cyclopropyl group).

Supplementary Table 7. Identified PEs from E. Coli extract by PB-MS^3. Both C=C and *sn*-positions were determined for each PE species (blue: *sn*-isomers).

PE	Precursor $[M+Na]^+$ (*m/z*)	Headgroup loss ion (*m/z*)	Diagnostic ions for *sn*-position (*m/z*)	Diagnostic ions for double bond position (*m/z*)
PE 14:0/16:1(9)	805.5	664.5	352, 368	461, 550
PE 16:1(9)/14:0	805.5	664.5	438	461, 550
PE 16:0/14:0	686.5	545.5	319	
PE 14:0/16:0	686.5	545.5	291	
PE 14:0/cy17:0	698.5	557.5	291	
PE 16:0/16:1(9)	833.5	692.5	352, 368	489, 578
PE 16:1(9)/16:0	833.5	692.5	438	489, 578
PE 16:0/cy17:0	726.5	585.5	319	
PE cy17:0/16:0	726.5	585.5	331	
PE 16:0/18:2(8,11)	859.5	718.5	378, 394	475, 564; 515, 604
PE 16:1(9)/18:1(11)	859.5	718.5	380, 396; 438	515, 604
PE 16:2(6,9)/18:0	859.6	718.5	436	475, 564; 515, 604
PE cy17:0/cy17:0	738.5	597.5	331	
PE 18:0/16:2(6,9)	859.5	718.5	350, 366	475, 564; 515, 604
PE 18:1(11)/16:1(9)	859.5	718.5	352, 368; 466	515, 604
PE 18:2(8,11)/16:0	859.5	718.5	464	475, 564; 515, 604
PE 16:0/18:1(11)	861.6	720.6	380, 396	517, 606
PE 18:1(11)/16:0	861.6	720.6	466	517, 606
PE 16:0/20:2(10,13)	887.6	746.6	406, 422	503, 592; 543, 632
PE cy17:0/cy19:0	766.5	625.5	331	
PE cy19:0/cy17:0	766.5	625.5	359	
PE 18:0/18:2(8,11)	887.6	746.6	378, 394	503, 592; 543, 632
PE 18:1(11)/18:1(11)	887.6	746.6	380, 396; 466	543, 632
PE 18:2(8,11)/18:0	887.6	746.6	464	503, 592; 543, 632

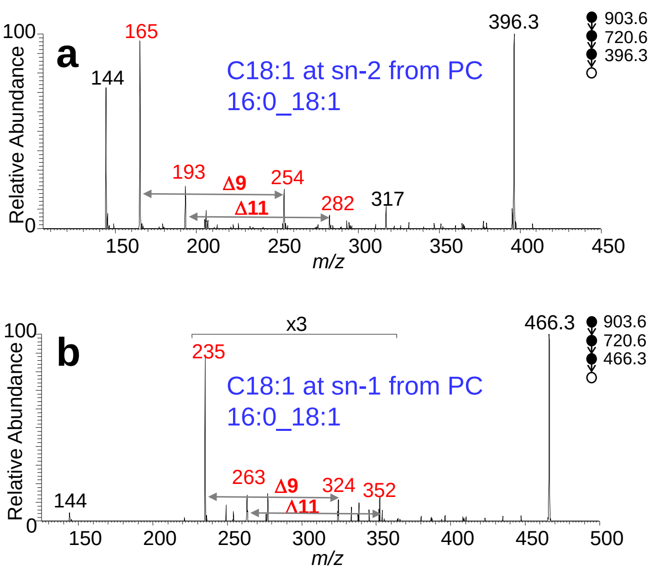

Supplementary Figure 16. Assignment of C=C location in individual fatty acyls at specific *sn*-positions in GPs. (a, b) MS^4 spectrum revealing C=C isomers of C18:1 cleaved from PC 16:0/18:1 (a) and PC 18:1/16:0 (b). (c, d) MS^4 spectrum revealing C=C isomers of C18:1 cleaved from PC 18:0/18:1 (c) and PC 18:1/18:0 (d). (Peaks labelled in red are C=C-specific diagnostic ions.)

Supplementary Note 2. Identification of different fragment ion groups.

In Figure 2a,b, MS signals at *m/z* 392, 394, 396, and 420 were *sn*-2-specific ions. These ions were here collected named as Group 1 (labelled blue). These MS signals can be readily assigned to corresponding fatty acyls using the fragmentation scheme in Figure 3. The set of MS signals at *m/z* 422, 424, 426, and 450 were actually sodiated PB products of FA 18:3, FA 18:2, FA 18:1, and FA 20:3, which are not *sn*-specific and named as Group 2 (labeled dark green). A set of MS signals that are 18 Da lower in mass (due to H_2O loss) than those in Group 2 were also detected, at *m/z* 404, 406, 408, and 432, which was named as Group 3 (labeled red). The relationship for these three groups of ions is shown in below (Supplementary Fig. 17). It is important to first determine which group a fragment belongs before the lipid structure characterization. In summary, Group 1 was *sn-2*-specific while Groups 2 and 3, which always accompany in a PB-MS^3 spectrum, are not.

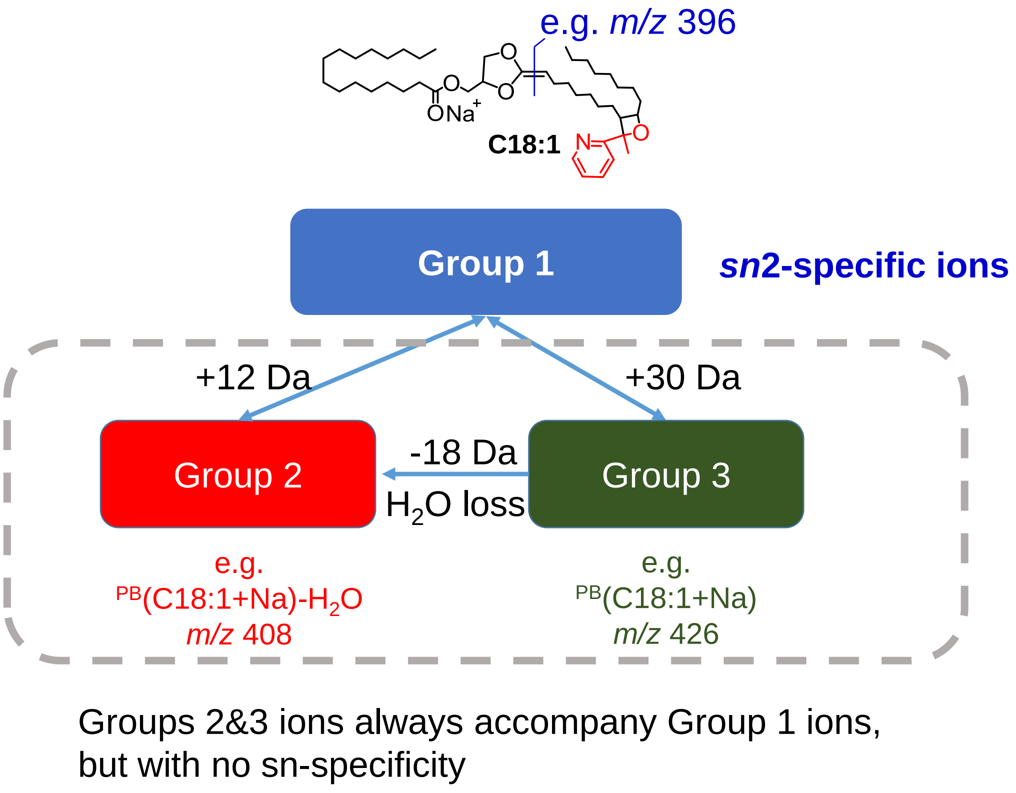

Supplementary Figure 17. Identification of different fragment ion groups in tandem MS spectra of isomeric mixtures.

Supplementary Table 8. Histological classification of breast cancer cell lines via the expression of estrogen receptor (ER), progesterone receptor (PR), and human epidermal growth factor receptor 2 (HER2).

Cell lines	ER<br>Estrogen receptor	PR<br>Progesterone receptor	HER2<br>Human epidermal growth factor receptor 2	Classification
MCF-7	+	+	–	Luminal A
BT-474	+	+	+	Luminal B
SK-BR-3	–	–	+	HER+
MDA-MB-468	–	–	–	Claudin-low

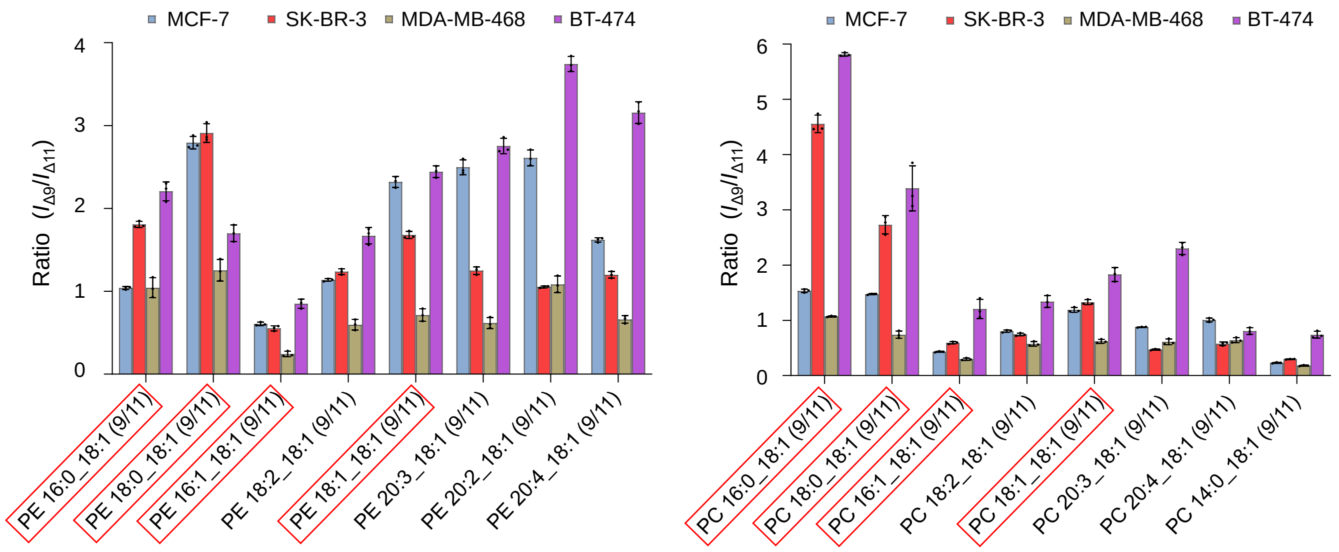

Supplementary Figure 18. Variations of C=C location isomer composition for C18:1-containing GPs in breast cancer cell lines studied. Those lipids emphasized in red rectangles were the most abundant ones. Error bar represents the standard deviation, $n = 3$. Source data is provided in a Source Data file.

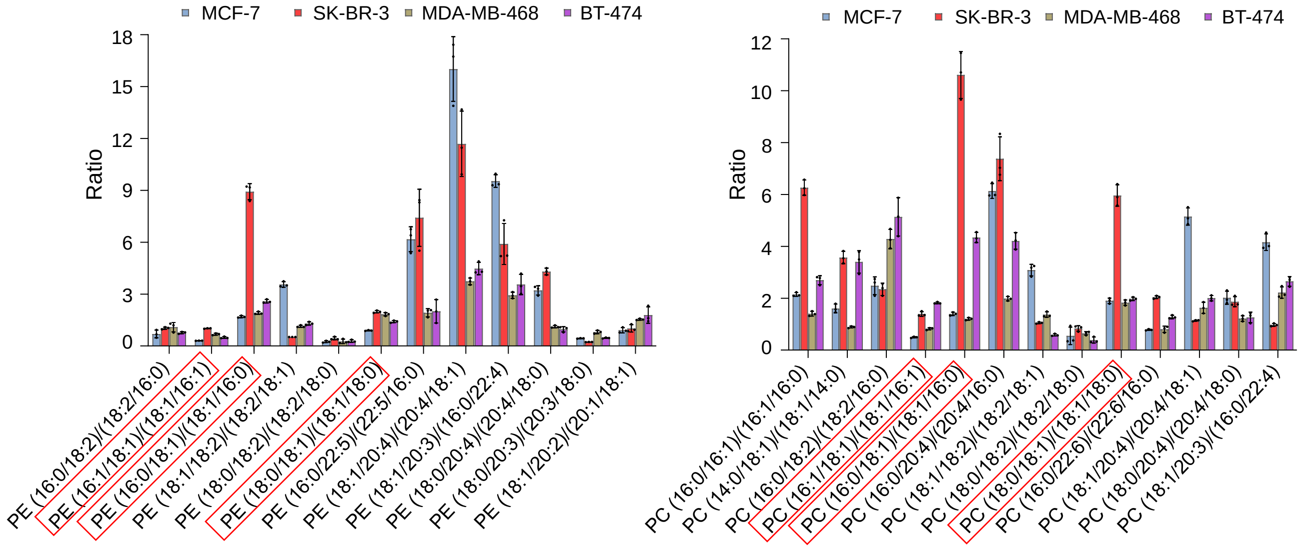

Supplementary Figure 19. Variations of *sn*-position isomer composition for GPs in breast cancer cell lines studied. Those lipids emphasized in red rectangles were the most abundant ones. Error bar represents the standard deviation, $n = 3$. Source data is provided in a Source Data file.

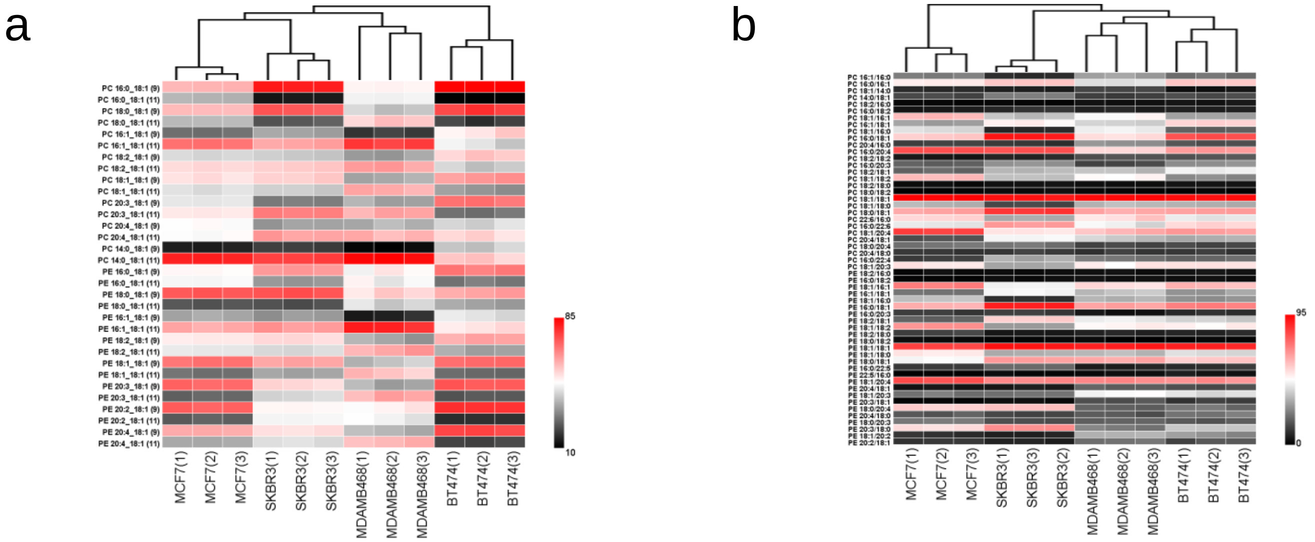

Supplementary Figure 20. Hierarchical cluster analysis discriminated the four subtypes of human breast cancer cells at the C=C location isomer level (a) and at the *sn*-position isomer level (b), respectively. Colors represent relative amounts as indicated by the color bar.

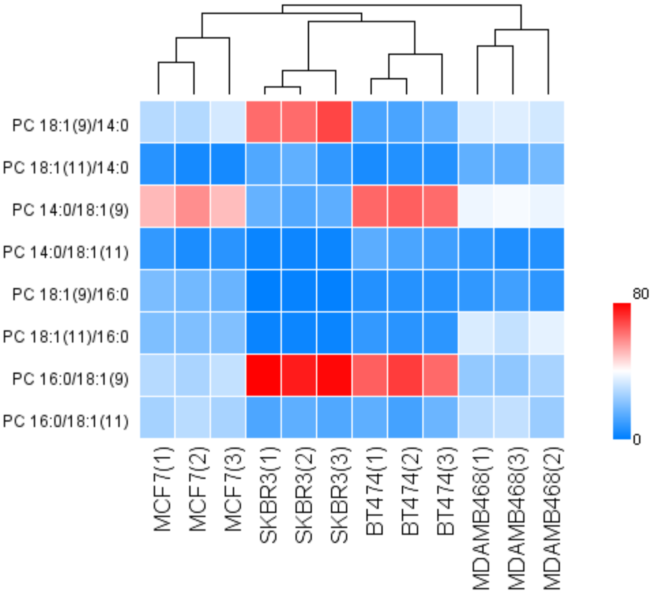

Supplementary Figure 21. Hierarchical cluster analysis discriminated the four subtypes of human breast cancer cells using C=C information specific for individual *sn*-positions. Colors represent relative amounts as indicated by the color bar.

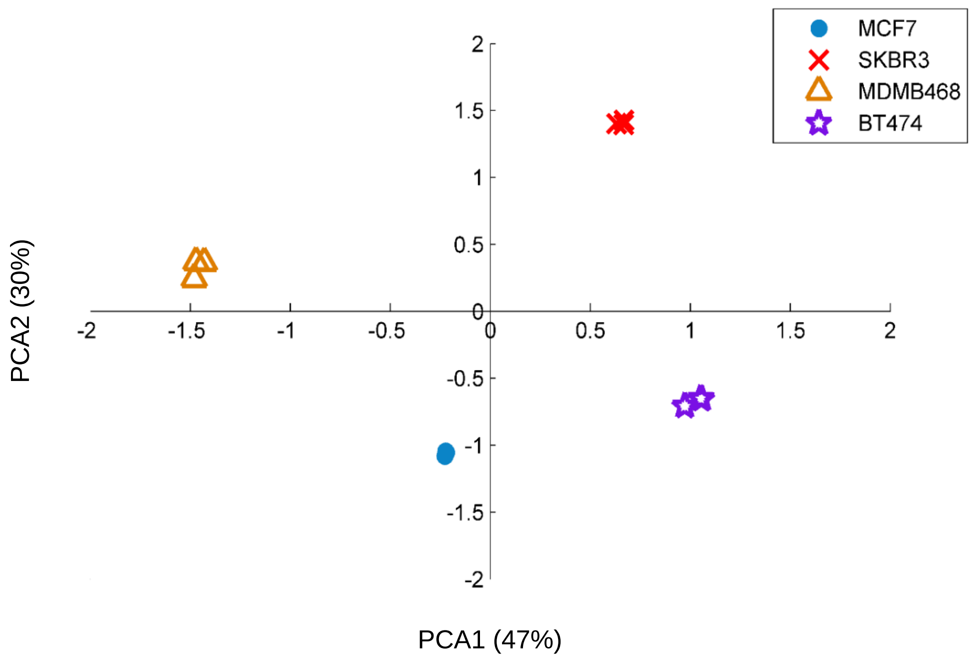

Supplementary Figure 22. Principal component analysis of four different human breast cancer subtypes.

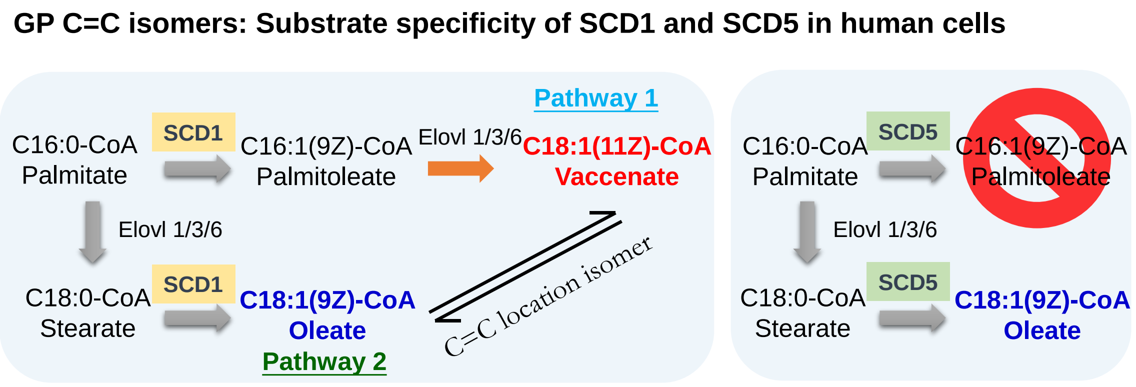

Supplementary Figure 23. Biosynthetic pathways for the synthesis of C18:1 (9) and (11) by stearoyl-CoA desaturases (SCDs) and elongases (Elovl 1/3/6). While SCD1 can desaturase both C16:0-CoA and C18:0-CoA, SCD5 can only desaturase C18:0-CoA to produce C18:1(9Z)-CoA.

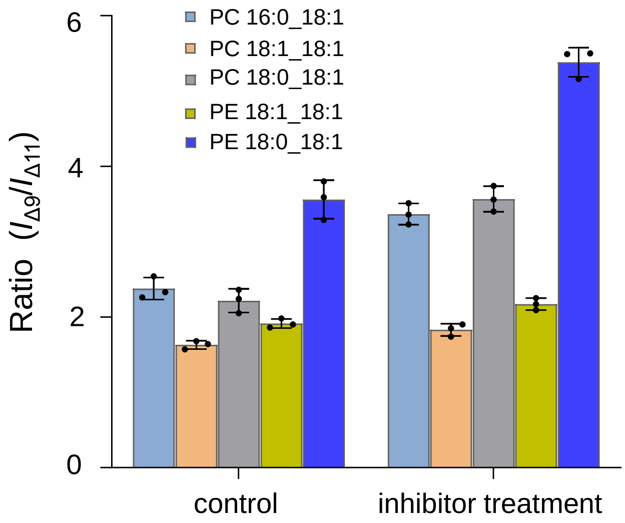

Supplementary Figure 24. Alternation of the C=C location isomer ratios (Δ9/Δ11) of C18:1-containing GPs using a SCD1 inhibitor (CAY10566, 10 nM) in MCF-7 cells. Error bar represents the standard deviation, $n = 3$. Source data is provided in a Source Data file.

Supplementary Table 9. The significant difference for the relative amount of PC species between each two breast cancer cell lines. The *p* values (calculated using two-tailed Student's *t* test) highlighted in red indicate no statistical difference.

Lipid species	MCF-7 VS SK-BR-3	MCF-7 VS MDA-MB-468	MCF-7 VS BT-474	SK-BR-3 VS MDA-MB-468	SK-BR-3 VS BT-474	MDA-MB-468 VS BT-474
PC 16:1/16:0	7.62396E-06	0.002770433	0.047117707	1.73158E-05	0.000124142	0.001591182
PC 16:0/16:1	4.73684E-05	5.5063E-05	5.53167E-05	5.1919E-06	0.151729677	5.41139E-06
PC 18:1/14:0	0.002902952	5.76706E-05	0.000619735	1.47664E-05	0.007004664	2.4684E-05
PC 14:0/18:1	6.90323E-05	0.039032124	0.357669086	4.16564E-06	3.50302E-05	0.007458144
PC 18:2/16:0	0.609048953	0.003502513	0.004864427	0.001591639	0.003392452	0.149104951
PC 16:0/18:2	0.015173921	0.096421332	0.056524677	0.084445727	0.043727358	0.721176942
PC 18:1/16:1	2.11831E-05	0.000347272	1.06004E-06	0.001119412	0.006314439	9.89683E-05
PC 16:1/18:1	4.03774E-05	0.000352318	3.67006E-06	0.000310981	0.003146273	9.8482E-06
PC 18:1/16:0	1.67563E-06	0.014098833	7.11105E-06	8.82804E-07	5.50335E-05	3.15457E-06
PC 16:0/18:1	1.67563E-06	0.014098833	7.11105E-06	8.82804E-07	5.50335E-05	3.15457E-06
PC 20:4/16:0	0.042355757	2.57974E-05	0.0188093	4.39995E-05	0.00578327	0.000148106
PC 16:0/20:4	0.222870882	1.51104E-07	7.12966E-06	3.86013E-07	1.26743E-05	3.24216E-05
PC 18:2/18:2	0.015582499	2.90618E-05	2.61664E-05	0.000298155	0.000245229	0.435728331
PC 16:0/20:3	9.62728E-05	0.002702083	0.00018075	4.10056E-05	0.607409448	6.32932E-05
PC 18:2/18:1	7.89381E-05	0.000275281	9.0918E-06	0.3690591	0.000132699	0.001721162
PC 18:1/18:2	4.98124E-07	9.2741E-05	2.31299E-06	3.34584E-05	0.000192202	1.96131E-05
PC 18:2/18:0	0.075418942	0.516559007	0.01467643	0.024661006	0.06055084	6.95079E-05
PC 18:0/18:2	0.021155655	0.222589117	0.726960685	0.026863473	0.02079196	0.302315353
PC 18:1/18:1	0.005510914	0.059093998	0.015729524	0.019042668	0.112861104	0.121887936
PC 18:1/18:0	2.15335E-05	0.435159348	0.341438636	2.02506E-05	8.05807E-06	0.105228308
PC 18:0/18:1	2.15335E-05	0.435159348	0.341438636	2.02506E-05	8.05807E-06	0.105228308
PC 22:6/16:0	2.0166E-06	0.844299902	0.000123752	0.000551703	0.000151214	0.007570817
PC 16:0/22:6	2.0166E-06	0.844299902	0.000123752	0.000551703	0.000151214	0.007570817
PC 18:1/20:4	7.03218E-07	0.00028862	3.52203E-05	0.008950626	5.09967E-05	0.061531565
PC 20:4/18:1	7.03218E-07	0.00028862	3.52203E-05	0.008950626	5.09967E-05	0.061531565
PC 18:0/20:4	0.863429651	0.000328721	0.000577959	0.000831177	0.001299815	0.570855528
PC 20:4/18:0	0.411455556	0.868740395	0.832702934	0.637654747	0.583658318	0.99709132
PC 16:0/22:4	7.77842E-05	0.000294679	0.001027922	0.002090124	0.000788123	0.059180004
PC 18:1/20:3	8.7197E-06	0.392730739	0.341724343	0.000360839	1.05407E-06	0.229088913
PC 16:0_18:1 (9)	9.03605E-07	1.27277E-05	1.37017E-07	8.40783E-08	0.000371091	2.28827E-09
PC 16:0_18:1 (11)	9.03605E-07	1.27277E-05	1.37017E-07	8.40783E-08	0.000371091	2.28827E-09
PC 18:0_18:1 (9)	3.81459E-05	0.000177763	0.00012534	2.86492E-05	0.044945627	3.76529E-05
PC 18:0_18:1 (11)	3.81459E-05	0.000177763	0.00012534	2.86492E-05	0.044945627	3.76529E-05
PC 16:1_18:1 (9)	0.000290298	0.00072114	0.000262365	8.23202E-05	0.001087002	0.000114059
PC 16:1_18:1 (11)	0.000290298	0.00072114	0.000262365	8.23202E-05	0.001087002	0.000114059
PC 18:2_18:1 (9)	0.02722465	0.001409382	0.000366908	0.00498411	0.000247188	0.000140673
PC 18:2_18:1 (11)	0.02722465	0.001409382	0.000366908	0.00498411	0.000247188	0.000140673
PC 18:1_18:1 (9)	0.02354131	7.15697E-05	0.000666699	3.45533E-05	0.001871369	2.58994E-05

PC 18:1_18:1 (11)	0.02354131	7.15697E-05	0.000666699	3.45533E-05	0.001871369	2.58994E-05
PC 20:3_18:1 (9)	7.94112E-07	0.001395346	3.05693E-06	0.006698382	5.40207E-07	1.49689E-05
PC 20:3_18:1 (11)	7.94112E-07	0.001395346	3.05693E-06	0.006698382	5.40207E-07	1.49689E-05
PC 20:4_18:1 (9)	0.000130939	0.000526235	0.007932768	0.108391729	0.003661035	0.019038113
PC 20:4_18:1 (11)	0.000130939	0.000526235	0.007932768	0.108391729	0.003661035	0.019038113
PC 14:0_18:1 (9)	0.000384457	0.002583791	4.17982E-05	0.0001065	8.67519E-05	2.74232E-05
PC 14:0_18:1 (11)	0.000384457	0.002583791	4.17982E-05	0.0001065	8.67519E-05	2.74232E-05

Supplementary Table 10. The significant difference for the relative amount of PE species between each two breast cancer cell lines. The *p* values (calculated using two-tailed Student's *t* test) highlighted in red indicate no statistical difference.

Lipid species	MCF-7 VS SK-BR-3	MCF-7 VS MDA-MB-468	MCF-7 VS BT-474	SK-BR-3 VS MDA-MB-468	SK-BR-3 VS BT-474	MDA-MB-468 VS BT-474
PE 18:2/16:0	0.637757933	0.295433032	0.051328575	0.282819093	0.01279396	0.093716446
PE 16:0/18:2	0.008375039	0.003770909	0.006928664	0.253112231	0.675765332	0.435386533
PE 18:1/16:1	1.27994E-06	5.78438E-05	0.000787776	0.000590907	0.000149963	0.00763354
PE 16:1/18:1	5.59504E-07	0.000229247	0.007370457	0.001322424	0.000342577	0.016646638
PE 18:1/16:0	1.0929E-06	0.022001156	0.000155613	2.68944E-06	4.984E-06	0.000842589
PE 16:0/18:1	1.0929E-06	0.022001156	0.000155613	2.68944E-06	4.984E-06	0.000842589
PE 16:0/20:3	0.635618064	0.000821345	0.039368073	0.000681792	0.024485233	0.003252124
PE 18:2/18:1	3.08583E-07	8.8434E-06	3.71878E-05	0.000110065	4.63378E-05	0.010067201
PE 18:1/18:2	2.09254E-07	4.81376E-05	0.000112847	1.89417E-05	4.33719E-05	0.825897753
PE 18:2/18:0	0.000241694	0.001309568	0.003462313	0.006629981	0.008949652	0.38325793
PE 18:0/18:2	0.10141528	0.099017064	0.062977144	0.495333106	0.775068022	0.600915652
PE 18:1/18:1	6.93328E-05	0.000450783	0.00064277	0.071116252	0.013550867	0.257245721
PE 18:1/18:0	6.36456E-06	3.19507E-05	0.000157101	0.102322146	0.000631145	0.003490105
PE 18:0/18:1	6.36456E-06	3.19507E-05	0.000157101	0.102322146	0.000631145	0.003490105
PE 16:0/22:5	2.43898E-06	0.00114799	0.180476011	2.04153E-05	0.000401927	0.036630777
PE 22:5/16:0	0.112250404	0.00657581	0.008856108	0.237542325	0.040442201	0.082395897
PE 18:1/20:4	9.78639E-05	0.000200509	0.0005138	0.780933097	0.254976198	0.232066878
PE 20:4/18:1	0.190671457	2.65859E-05	5.45011E-05	7.87273E-05	0.000207937	0.008209041
PE 18:1/20:3	0.665731051	6.45223E-05	0.000236436	8.06442E-06	4.95753E-05	0.018403031
PE 20:3/18:1	0.025057438	5.06996E-05	0.000351723	0.000115919	0.000892466	0.019884109
PE 18:0/20:4	0.010735311	1.0159E-06	1.73192E-05	1.55024E-07	8.88002E-06	0.077351472
PE 20:4/18:0	0.01830639	0.662383624	0.012838385	0.036597971	0.000524972	0.00950863
PE 18:0/20:3	2.34404E-05	0.000749656	0.00091938	0.001198711	0.060608334	0.074701061
PE 20:3/18:0	7.78064E-06	1.4411E-05	2.56823E-06	2.66642E-06	5.78586E-08	0.000260985
PE 18:1/20:2	0.021837472	3.97186E-06	0.000413318	6.00971E-07	0.000197937	0.018258153
PE 20:2/18:1	0.003699555	6.84046E-05	0.033153577	3.14386E-05	0.005122898	0.018513234
PE 16:0_18:1 (9)	3.36241E-06	0.972999105	1.43371E-05	0.001438658	0.003475685	0.000602562
PE 16:0_18:1 (11)	3.36241E-06	0.972999105	1.43371E-05	0.001438658	0.003475685	0.000602562
PE 18:0_18:1 (9)	0.191758104	0.000274033	0.00023546	0.000248874	0.000220866	0.011949414
PE 18:0_18:1 (11)	0.191758104	0.000274033	0.00023546	0.000248874	0.000220866	0.011949414
PE 16:1_18:1 (9)	0.071624658	0.000118632	0.001898909	0.000277194	0.001105785	5.74707E-05
PE 16:1_18:1 (11)	0.071624658	0.000118632	0.001898909	0.000277194	0.001105785	5.74707E-05
PE 18:2_18:1 (9)	0.007035908	0.000466026	0.000360542	0.000310613	0.001318756	0.000121576
PE 18:2_18:1 (11)	0.007035908	0.000466026	0.000360542	0.000310613	0.001318756	0.000121576
PE 18:1_18:1 (9)	0.000147575	4.56299E-05	0.100019915	0.000145818	8.20103E-05	3.92173E-05

PE 18:1_18:1 (11)	0.000147575	4.56299E-05	0.100019915	0.000145818	8.20103E-05	3.92173E-05
PE 20:3_18:1 (9)	2.17492E-05	2.41505E-05	0.024991463	0.000349433	1.19741E-05	1.87158E-05
PE 20:3_18:1 (11)	2.17492E-05	2.41505E-05	0.024991463	0.000349433	1.19741E-05	1.87158E-05
PE 20:2_18:1 (9)	1.18661E-06	0.000128201	0.000147982	0.664955553	5.86269E-08	3.7123E-05
PE 20:2_18:1 (11)	1.18661E-06	0.000128201	0.000147982	0.664955553	5.86269E-08	3.7123E-05
PE 20:4_18:1 (9)	0.000113963	3.03937E-05	9.39039E-06	0.000184915	4.14608E-06	5.47573E-06
PE 20:4_18:1 (11)	0.000113963	3.03937E-05	9.39039E-06	0.000184915	4.14608E-06	5.47573E-06

Supplementary Table 11. The significant difference of the intensity ratio for each set of lipid structural isomers between each two breast cancer cell lines. The *p* values (calculated using two-tailed Student's *t* test) highlighted in red indicate no statistical difference.

Intensity ratio	MCF-7 VS SK-BR-3	MCF-7 VS MDA-MB-468	MCF-7 VS BT-474	SK-BR-3 VS MDA-MB-468	SK-BR-3 VS BT-474	MDA-MB-468 VS BT-474
PC (16:0/16:1)/(16:1/16:0)	2.02664E-05	0.000416279	0.007353872	1.10578E-05	5.75049E-05	0.000343311
PC (14:0/18:1)/(18:1/14:0)	0.000299424	0.001941098	0.002639299	3.95223E-05	0.591116312	0.000563237
PC (16:0/18:2)/(18:2/16:0)	0.609048953	0.003502513	0.004864427	0.001591639	0.003392452	0.149104951
PC (16:1/18:1)/(18:1/16:1)	9.24449E-05	0.00044745	8.80756E-07	0.000718915	0.001902793	8.67485E-06
PC (16:0/18:1)/(18:1/16:0)	6.1801E-05	0.014551806	1.58803E-05	5.66597E-05	0.000304249	1.13817E-05
PC (16:0/20:4)/(20:4/16:0)	0.073128931	1.77662E-05	0.001493673	0.000384273	0.003698814	0.000312294
PC (18:1/18:2)/(18:2/18:1)	0.000100983	0.000281897	4.51731E-05	0.007576951	8.99055E-05	0.000269003
PC (18:0/18:2)/(18:2/18:0)	0.23345776	0.648101125	0.475635426	0.062056592	0.007726622	0.026017017
PC (18:0/18:1)/(18:1/18:0)	7.77734E-05	0.43333096	0.342664454	7.22264E-05	7.79266E-05	0.101181334
PC (16:0/22:6)/(22:6/16:0)	3.85273E-06	0.786742155	0.000230871	9.04248E-05	0.000102232	0.003967755
PC (18:1/20:4)/(20:4/18:1)	2.99689E-05	9.94221E-05	9.48826E-05	0.016552443	0.000157972	0.051743965
PC (18:0/20:4)/(20:4/18:0)	0.44032143	0.006822587	0.014778066	0.007271379	0.020290559	0.748762461
PC (18:1/20:3)/(16:0/22:4)	7.447E-05	0.001029793	0.002201415	0.000694516	0.000112603	0.060798639
PC 16:0_18:1 (9/11)	5.77831E-06	2.24215E-05	7.75527E-09	3.04553E-06	0.000186557	1.23895E-09
PC 18:0_18:1 (9/11)	0.000173345	4.78995E-05	0.001277257	3.84194E-05	0.060232237	0.000385102
PC 16:1_18:1 (9/11)	0.000361612	0.000579996	0.001437165	7.5296E-05	0.003552895	0.0007952
PC 18:2_18:1 (9/11)	0.026430501	0.001011692	0.000897058	0.004285544	0.000617577	0.000287898
PC 18:1_18:1 (9/11)	0.024200218	7.03522E-05	0.001209623	3.16377E-05	0.002949771	9.14362E-05
PC 20:3_18:1 (9/11)	5.07897E-07	0.000853335	2.59469E-05	0.00926707	9.59977E-06	1.88244E-05
PC 20:4_18:1 (9/11)	9.65666E-05	0.000371838	0.006541623	0.109789698	0.004338331	0.020215399
PC 14:0_18:1 (9/11)	0.000366933	0.002438105	0.000155527	8.68323E-05	0.000270317	0.000107788
PE (16:0/18:2)/(18:2/16:0)	0.070163987	0.127999204	0.623008057	0.739063118	0.005922416	0.116679627
PE (16:1/18:1)/(18:1/16:1)	4.86382E-08	0.000298057	0.005471823	0.000465103	8.49135E-05	0.012322229
PE (16:0/18:1)/(18:1/16:0)	1.2385E-05	0.023949429	0.00020785	1.434E-05	2.2177E-05	0.000888271
PE (18:1/18:2)/(18:2/18:1)	4.64751E-06	1.54237E-05	2.86529E-05	7.02308E-05	0.000146257	0.066613909
PE (18:0/18:2)/(18:2/18:0)	0.030944731	0.847670972	0.522012257	0.125301629	0.086589985	0.816075361
PE (18:0/18:1)/(18:1/18:0)	1.3612E-05	9.4871E-05	0.000268713	0.098092207	0.000518349	0.003801951
PE (16:0/22:5)/(22:5/16:0)	0.296781778	0.000613314	0.001841119	0.004646432	0.006269956	0.84593865
PE (18:1/20:4)/(20:4/18:1)	0.048082409	0.000348423	0.000466478	0.001936409	0.002922777	0.034718593

PE (18:1/20:3)/(16:0/22:4)	0.007155228	9.74754E-06	0.000117354	0.012920689	0.037852685	0.15276773
PE (18:0/20:4)/(20:4/18:0)	0.00456259	0.000192727	0.000220146	1.20569E-05	1.96106E-05	0.204637953
PE (18:0/20:3)/(20:3/18:0)	1.53358E-05	0.002386804	0.252487186	0.000417381	0.000161736	0.003509412
PE (18:1/20:2)/(20:1/18:1)	0.531072077	0.002238926	0.037989507	0.015251246	0.062499855	0.411612637
PE 16:0_18:1 (9/11)	5.81582E-06	0.976531841	6.55721E-05	0.000508087	0.004695444	0.00028225
PE 18:0_18:1 (9/11)	0.197278036	5.99645E-05	0.000113639	7.64016E-05	0.000156288	0.009788931
PE 16:1_18:1 (9/11)	0.069858354	7.9254E-05	0.002543682	0.000242469	0.00139387	8.34512E-05
PE 18:2_18:1 (9/11)	0.007657968	0.000160462	0.000751233	0.000113471	0.001995975	9.80081E-05
PE 18:1_18:1 (9/11)	0.000171804	1.00174E-05	0.099050473	4.26868E-05	0.000100072	8.26907E-06
PE 20:3_18:1 (9/11)	2.84421E-05	7.94643E-06	0.025869588	0.000180943	1.53371E-05	5.30432E-06
PE 20:2_18:1 (9/11)	8.96051E-06	4.31823E-05	0.000106802	0.63609032	7.73774E-07	4.20647E-06
PE 20:4_18:1 (9/11)	8.46838E-05	7.45297E-06	3.88331E-05	0.000102219	1.57322E-05	6.65529E-06

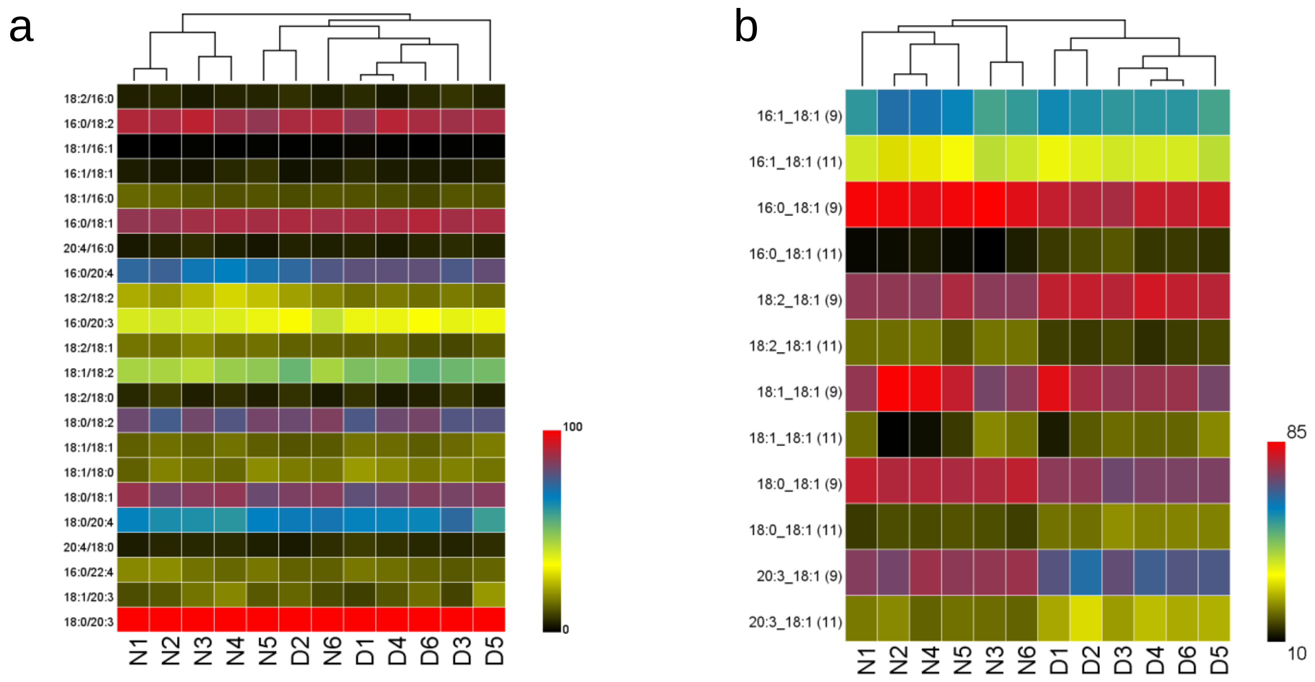

Supplementary Figure 25. Hierarchical cluster analysis of normal (N1-N6) and T2D (D1-D6) human plasma samples (a) using 22 *sn*-position isomers and (b) using 12 C=C location isomers, respectively. Colors represent relative amounts as indicated by the color bar.

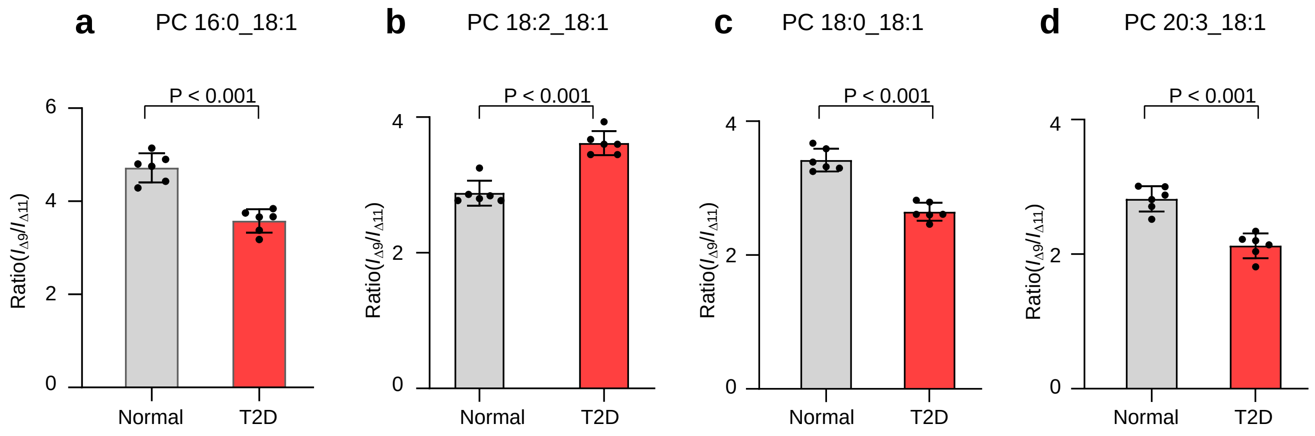

Supplementary Figure 26. Relative C=C location isomer ratios ($\Delta 9/\Delta 11$) from PCs containing C18:1. (a) PC 16:0_18:1, (b) PC 18:2_18:1, (c) PC 18:0_18:1, and (d) PC 20:3_18:1. *P* values were generated by two-tailed student's *t* test. Error bar represents the standard deviation, $n = 6$. Source data is provided in a Source Data file.

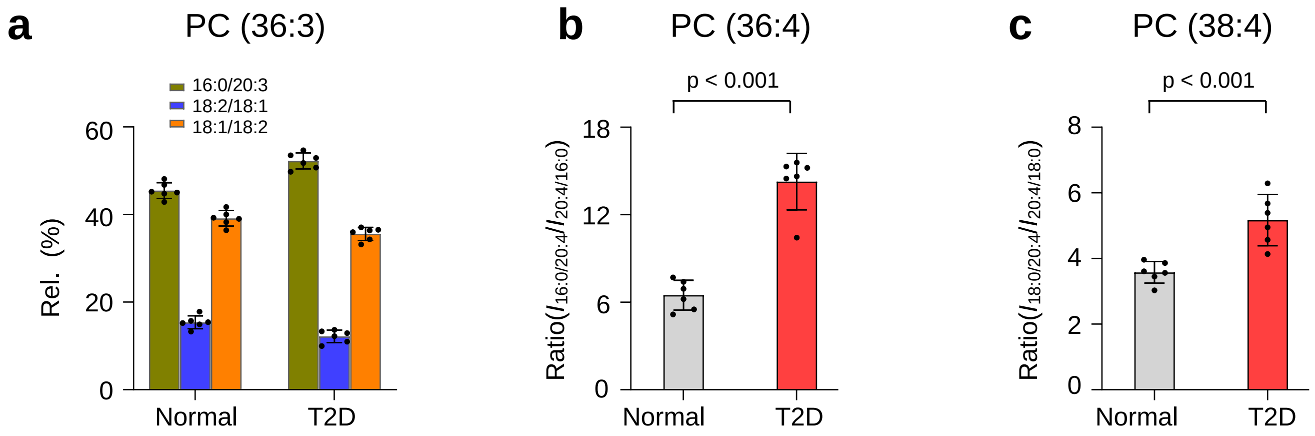

Supplementary Figure 27. The representative *sn*-isomers of PCs. (a) Relative abundance of *sn*-position isomers of PC 36:3. Relative ratios of *sn*-position isomers of (b) PC 36:4 and (c) PC 38:4 in normal and T2D human plasma samples. *P* values were generated by two-tailed student's *t* test. Error bar represents the standard deviation, *n* = 6. Source data is provided in a Source Data file.

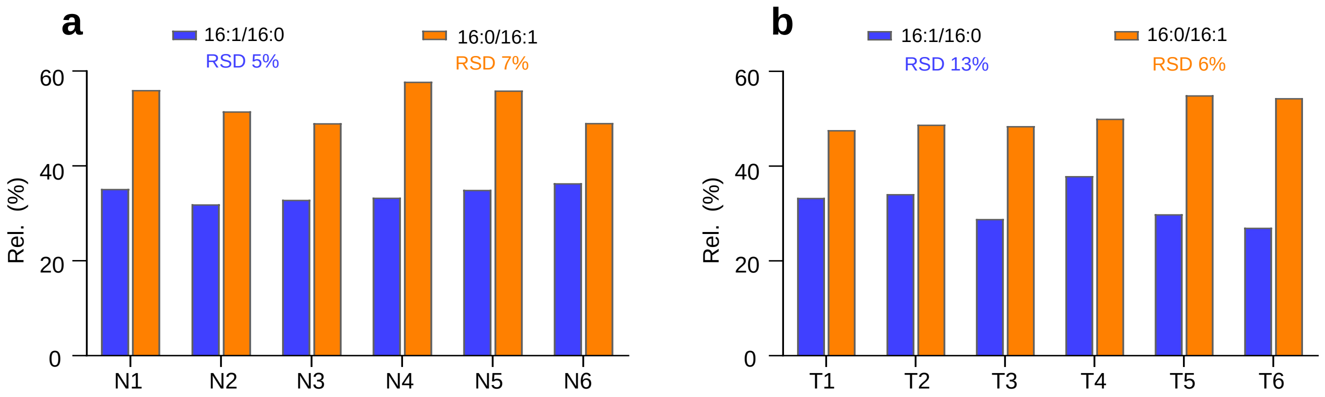

Supplementary Figure 28. Variations in the relative amounts of *sn*-position isomers (PC 16:0_16:1) in (a) 6 paracarcinoma tissues and (b) 6 corresponding lung cancer tissues.

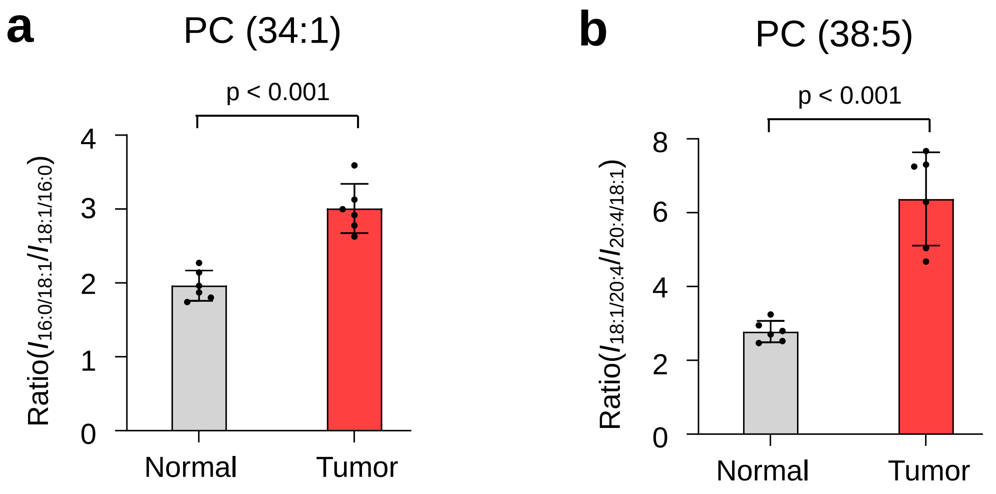

Supplementary Figure 29. The relative ratios of *sn*-position isomers of (a) PC 34:1 and (b) PC 38:5 in the paracarcinoma and lung cancer tissues. *P* values were generated by two-tailed student's *t* test. Error bar represents the standard deviation, $n = 6$. Source data is provided in a Source Data file.

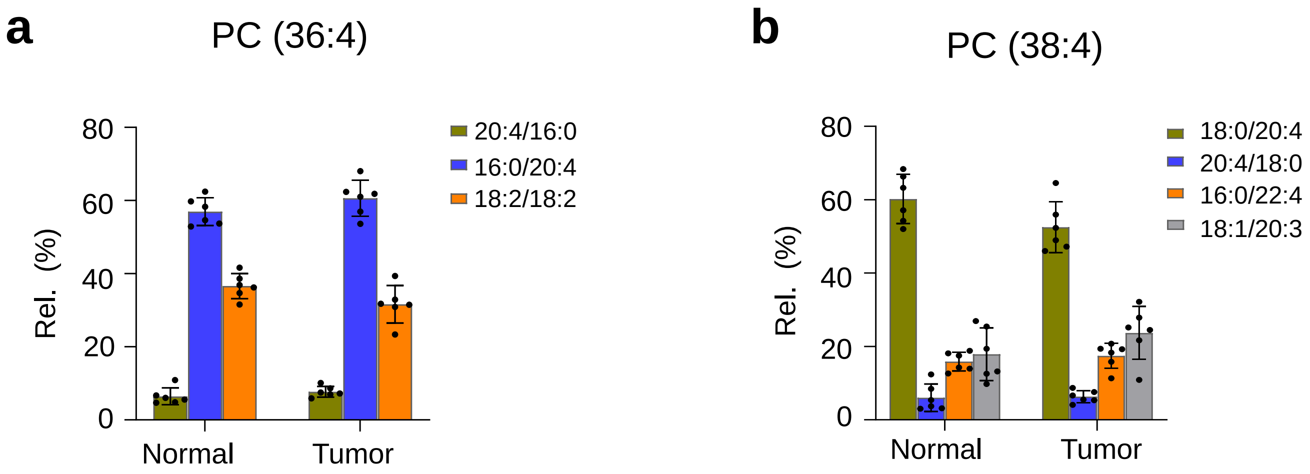

Supplementary Figure 30. The relative abundance of *sn*-position isomers of (a) PC 36:4 and (b) PC 38:4 in the paracarcinoma and lung cancer tissues. Error bar represents the standard deviation, $n = 6$. Source data is provided in a Source Data file.

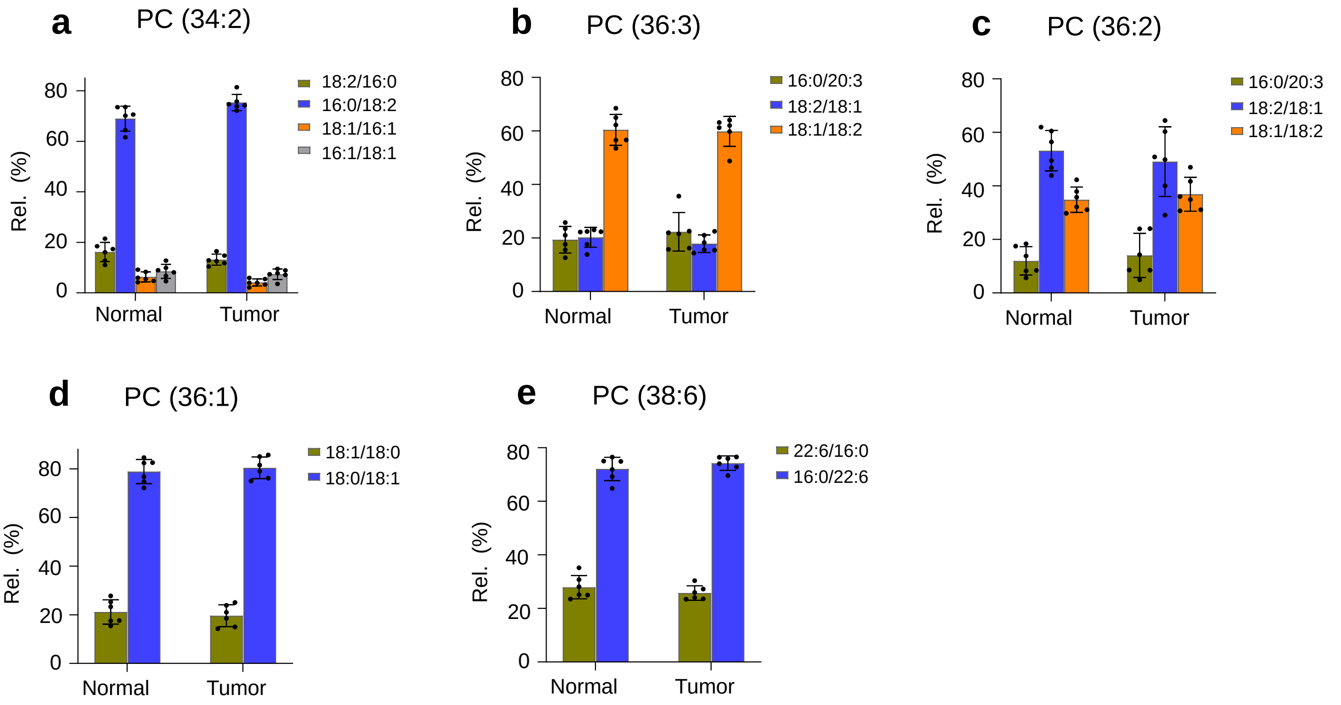

Supplementary Figure 31. The relative abundance of *sn*-position isomers of (a) PC 34:2, (b) PC 36:3, (c) PC 36:2, (d) PC 36:1, and (e) PC 38:6 in paracarcinoma and lung cancer tissues. Error bar represents the standard deviation, n = 6. Source data is provided in a Source Data file.

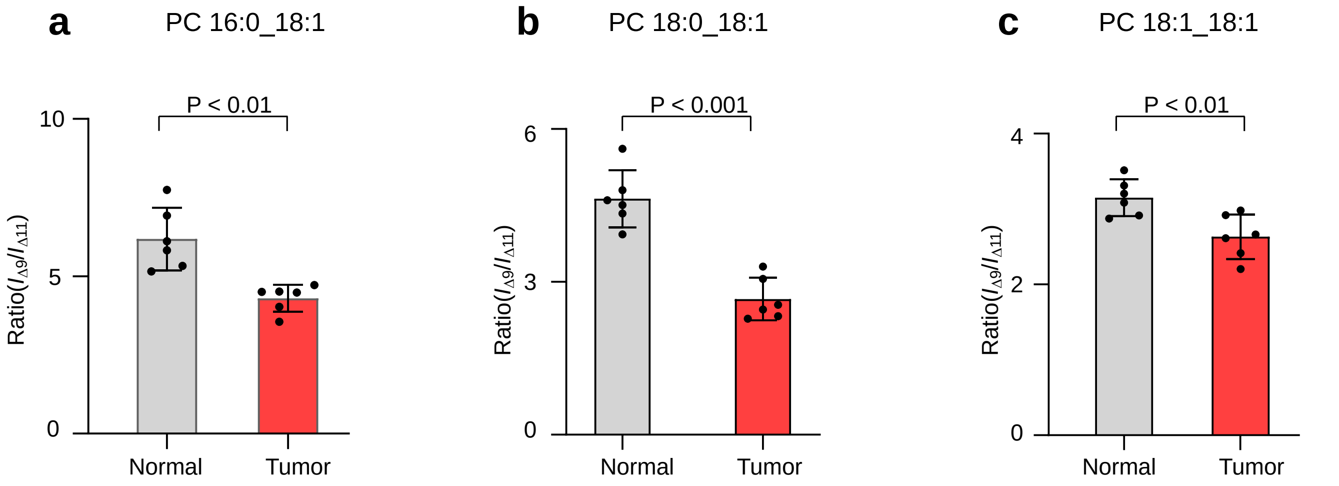

Supplementary Figure 32. The C=C location isomer ratios ($I_{\Delta 9}/I_{\Delta 11}$) of (a) PC 34:1, (b) PC 36:1 and (c) PC 36:2 in paracarcinoma and lung cancer tissues. P values were generated by two-tailed student's t test. Error bar represents the standard deviation, n = 6. Source data is provided in a Source Data file.

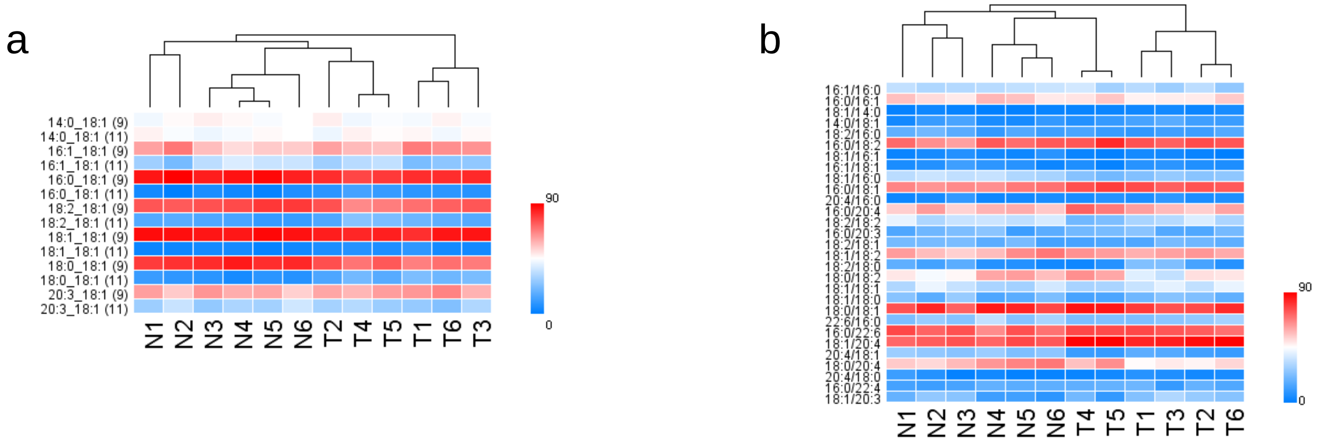

Supplementary Figure 33. Hierarchical cluster analysis of paired human NSCLC (T1-T6) and adjacent normal tissues (N1-N6) (a) using 14 C=C location isomers and (b) using 29 *sn*-isomers, respectively. Colors represent relative amounts as indicated by the color bar.

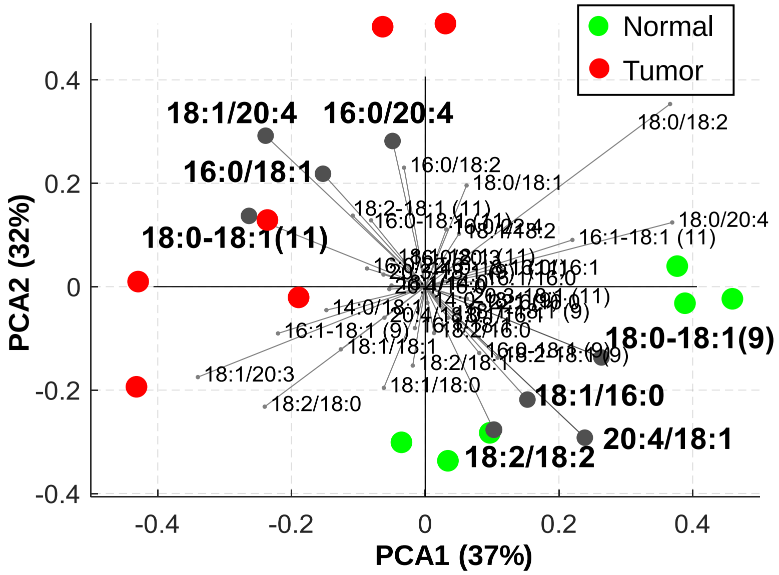

Supplementary Figure 34. Principal component analysis and loading plot of human NSCLC and adjacent normal tissues, revealing prominent structurally defined lipids contributing most to classification.

Supplementary Note 3. PB-MS^3 analysis on a high resolution mass spectrometer.

The method had been tested on a high resolution MS, i.e. Thermo LTQ Orbitrap Velos Pro mass spectrometer. The pair of lipid *sn*-isomers tested was PC 16:0/18:1(9Z) and PC 18:1(9Z)/16:0. The analytical workflow is the same as that performed on a low resolution MS, except that the mass resolution (60,000) for both precursor and fragment ions are higher. The MS^3 spectrum acquired using ion trap CID was shown in the Supplementary Fig. 35, with abundant diagnostic ions clearly detected. The diagnostics ions and their corresponding high resolution *m/z* values (mass error < 2 ppm) was listed in the Supplementary Table 12.

The instrument parameters used were as follows: nanoESI voltage, +1600 V; capillary temperature, 270 ℃; lens 1 voltage, -15 V; front lens voltage, -10 V; maximum injection time, 200 ms; microscans, 3; isolation width, 1 Th; normalized collision energy (NCE) settings of MS^2 by CID, 45 a.u.; NCE settings of MS^3 by CID, 35 a.u.

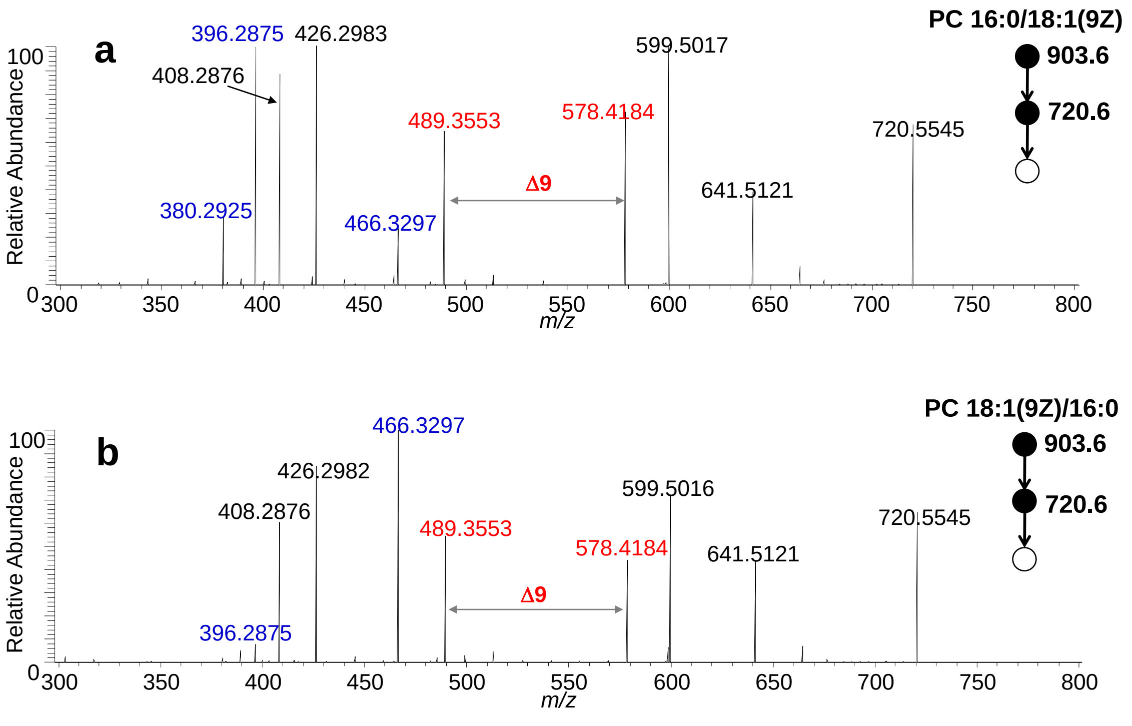

Supplementary Figure 35. The PB-MS^3 spectra of PC 16:0/18:1(9Z) (a) and PC 18:1(9Z)/16:0 (b) using high resolution MS. (Peaks labelled in red and blue/purple are C=C- and *sn*-specific diagnostic ions, respectively.)

Supplementary Table 12. Comparison of the *m/z* values of calculation and measurement.

Diagnostic ions	Calculated (*m/z*)	Measured (*m/z*)	Mass error (ppm)
Precursor 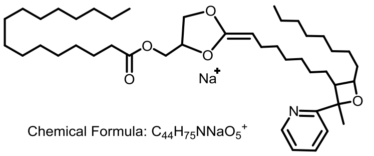	720.5537	720.5545	1.1
sn diagnostic ions 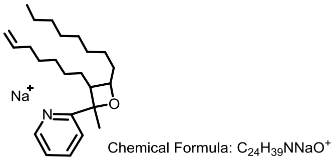	380.2924	380.2925	0.3
sn diagnostic ions 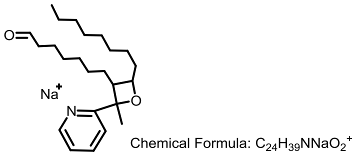	396.2873	396.2875	0.5
sn diagnostic ions 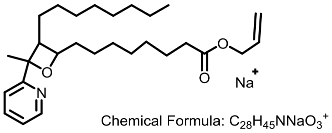	466.3292	466.3297	1.1
C=C diagnostic ions 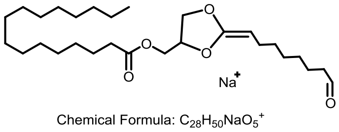	489.3550	489.3553	0.6
C=C diagnostic ions 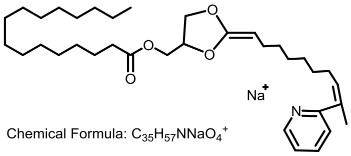	578.4180	578.4184	0.7
