# Supplementary material for: Large-scale lipid analysis with C=C location and sn-position isomer resolving power
Source: Nat Commun. 2020 Jan 17;11:375. doi: 10.1038/s41467-019-14180-4 (PMC6969141; doi:10.1038/s41467-019-14180-4)
Supplement: Supplementary file 2 — Reporting Summary [file 41467_2019_14180_MOESM2_ESM.pdf]

nature research

Corresponding author(s): Zheng Ouyang, Xiaoxiao Ma

Last updated by author(s): Dec 11, 2019

Reporting Summary

Nature Research wishes to improve the reproducibility of the work that we publish. This form provides structure for consistency and transparency in reporting. For further information on Nature Research policies, see Authors & Referees and the Editorial Policy Checklist .

Statistics

For all statistical analyses, confirm that the following items are present in the figure legend, table legend, main text, or Methods section.

n/a	Confirmed	
☐	☒	The exact sample size (*n*) for each experimental group/condition, given as a discrete number and unit of measurement
☐	☒	A statement on whether measurements were taken from distinct samples or whether the same sample was measured repeatedly
☐	☒	The statistical test(s) used AND whether they are one- or two-sided<br>*Only common tests should be described solely by name; describe more complex techniques in the Methods section.*
☒	☐	A description of all covariates tested
☒	☐	A description of any assumptions or corrections, such as tests of normality and adjustment for multiple comparisons
☐	☒	A full description of the statistical parameters including central tendency (e.g. means) or other basic estimates (e.g. regression coefficient) AND variation (e.g. standard deviation) or associated estimates of uncertainty (e.g. confidence intervals)
☐	☒	For null hypothesis testing, the test statistic (e.g. *F*, *t*, *r*) with confidence intervals, effect sizes, degrees of freedom and *P* value noted<br>*Give P values as exact values whenever suitable.*
☒	☐	For Bayesian analysis, information on the choice of priors and Markov chain Monte Carlo settings
☐	☒	For hierarchical and complex designs, identification of the appropriate level for tests and full reporting of outcomes
☒	☐	Estimates of effect sizes (e.g. Cohen's *d*, Pearson's *r*), indicating how they were calculated

Our web collection on statistics for biologists contains articles on many of the points above.

Software and code

Policy information about availability of computer code

Data collection	Analyst 1.6.3 from Sciex, Xcalibur 2.2 from Thermo
Data analysis	Analyst 1.6.3 from Sciex, Xcalibur 2.2 from Thermo, HemI software (version 1.0, http://hemi.biocuckoo.org/down.php) was used for hierarchical cluster analysis, Principal component analysis was performed using Matlab (version R2014b).

For manuscripts utilizing custom algorithms or software that are central to the research but not yet described in published literature, software must be made available to editors/reviewers. We strongly encourage code deposition in a community repository (e.g. GitHub). See the Nature Research guidelines for submitting code & software for further information.

Data

Policy information about availability of data

All manuscripts must include a data availability statement. This statement should provide the following information, where applicable:

- Accession codes, unique identifiers, or web links for publicly available datasets
- A list of figures that have associated raw data
- A description of any restrictions on data availability

Data generated or analysed during this study are included in this article its supplementary information files.
All data are available from the corresponding author upon reasonable request.
The source data for Figs. 1e, 3c, 4b–d, 5c and Supplementary Figs. 18, 19, 24, 26, 27, 29-32, and Supplementary Table 4 are provided in a Source Data file.

Field-specific reporting

Please select the one below that is the best fit for your research. If you are not sure, read the appropriate sections before making your selection.

Life sciences study design

All studies must disclose on these points even when the disclosure is negative.

Sample size	We used 4 kinds of breast cancer cell lines, 6 normal plasma samples and 6 type 2 diabetes plasma samples, 6 non-small cell lung cancer and 6 adjacent normal tissue samples. These samples were just used to demonstrate the possible application of the developed lipidomic approach, but not for discover the actual biomarkers. For cell lines experiment, sample size of 3 was used to reach meaningful conclusions of the data. For plasma and tissue samples experiment, sample size of 6 was show a relative reproducibility of the lipid analysis results.
Data exclusions	No data were excluded.
Replication	Experimental findings were reliably reproduced.
Randomization	For cell lines experiment, samples were grouped into 4 kinds of cell lines. For plasma and tissue samples experiment, samples were grouped into normal/type 2 diabetes samples, and normal/lung cancer tissue samples according to the diagnosis results. No further grouping was applied.
Blinding	Blinding was not applied., because the demonstrations in breast cancer cell lines, normal/type 2 diabetes samples, and normal/lung cancer samples were just to show the difference between different groups of samples.

Reporting for specific materials, systems and methods

We require information from authors about some types of materials, experimental systems and methods used in many studies. Here, indicate whether each material, system or method listed is relevant to your study. If you are not sure if a list item applies to your research, read the appropriate section before selecting a response.

Materials & experimental systems

n/a	Involved in the study	
☒	☐	Antibodies
☐	☒	Eukaryotic cell lines
☒	☐	Palaeontology
☒	☐	Animals and other organisms
☐	☒	Human research participants
☒	☐	Clinical data

Methods

n/a	Involved in the study	
☒	☐	ChIP-seq
☒	☐	Flow cytometry
☒	☐	MRI-based neuroimaging

Eukaryotic cell lines

Policy information about cell lines

Cell line source(s)	Human breast cancer cell lines including MCF7 (catalogue number 3111C0001CCC000013), SK-BR-3 (catalogue number 3111C0001CCC000085), BT-474 (catalogue number 3111C0001CCC000129) and MDA-MB-468 (catalogue number 3111C0001CCC000249), were obtained from National Infrastructure of Cell Line Resource (Beijing, China).
Authentication	Cell lines were authenticated by short tandem repeat (STR) prior to purchase.
Mycoplasma contamination	The cell lines were not tested for mycoplasma contamination.
Commonly misidentified lines (See ICLAC register)	No commonly misidentified lines used.

Human research participants

Policy information about studies involving human research participants

Population characteristics	Samples were obtained from specimen bank of the hospital. All the samples were from Asian individuals. Normal plasma were from healthy individuals at the age of 41-65. Type 2 diabetes plasma were from individuals at the age of 42-78. Non-small cell lung cancer and adjacent normal tissue samples were from individuals at the age of 46-75, including man and women patients.
Recruitment	No relevant. Samples were obtained from specimen bank of the hospital.
Ethics oversight	The study protocol was approved by the Ethical Review Board of Tsinghua University.

Note that full information on the approval of the study protocol must also be provided in the manuscript.
